# A New Framework for Understanding Recombination-Limited Charge Extraction in Disordered Semiconductors

**DOI:** 10.1021/acs.jpclett.4c00218

**Published:** 2024-04-16

**Authors:** Austin M. Kay, Drew B. Riley, Paul Meredith, Ardalan Armin, Oskar J. Sandberg

**Affiliations:** † Sustainable Advanced Materials (Sêr-SAM), Centre for Integrative Semiconductor Materials (CISM), Department of Physics, Swansea University Bay Campus, Swansea SA1 8EN, United Kingdom; ‡ Physics, Faculty of Science and Engineering, Åbo Akademi University, 20500 Turku, Finland

## Abstract

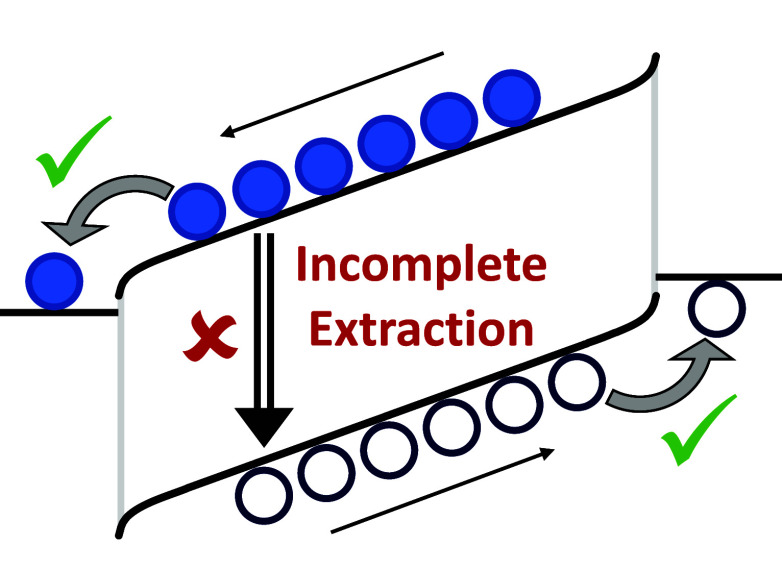

Recombination of free charges is a key loss mechanism limiting the performance of organic semiconductor-based photovoltaics such as solar cells and photodetectors. The carrier density-dependence of the rate of recombination and the associated rate coefficients are often estimated using transient charge extraction (CE) experiments. These experiments, however, often neglect the effect of recombination during the transient extraction process. In this work, the validity of the CE experiment for low-mobility devices, such as organic semiconductor-based photovoltaics, is investigated using transient drift-diffusion simulations. We find that recombination leads to incomplete CE, resulting in carrier density-dependent recombination rate constants and overestimated recombination orders; an effect that depends on both the charge carrier mobilities and the resistance–capacitance time constant. To overcome this intrinsic limitation of the CE experiment, we present an analytical model that accounts for charge carrier recombination, validate it using numerical simulations, and employ it to correct the carrier density-dependence observed in experimentally determined bimolecular recombination rate constants.

Photovoltaic devices based on next-generation, solution-processable organic semiconductors are—at the time of writing—edging toward commercial viability, particularly in applications such as indoor light harvesting and building-integrated photovoltaics, in which requirements such as band gap tunability are important considerations. With current state-of-the-art power conversion efficiencies (PCEs) of organic solar cells surpassing 19% now regularly reported in the literature,^1−4^ minor improvements in device-level and material-level characteristics could pave the way to PCEs above and beyond 20%.^5^ Of the several loss mechanisms that currently restrain organic photovoltaics (OPVs), charge carrier recombination of photogenerated electrons and holes is a major limiting factor,^6^ giving rise to losses in both the open-circuit voltage and the fill factor.

There exists a repertoire of transient electrical techniques for probing charge carrier recombination in OPVs,^6−15^ including transient photovoltage (TPV), charge extraction by linearly increasing voltage (CELIV), time-delayed collection field (TDCF), and transient charge extraction (CE) experiments. CE experiments have, in particular, been widely used to infer the carrier density (*n*) dependence of the recombination rate  in OPVs. It is generally expected that the recombination rate in OPVs is of a bimolecular form , ideally characterized by a carrier density-independent recombination rate constant β and reaction order δ = 2.^16^ Despite this, CE experiments have frequently suggested a carrier density-dependence in β that leads to δ > 2, which has commonly been attributed to recombination via trap-like tail states.^17−20^ It was recently shown, however, that most electrical techniques (including CE) are detrimentally affected by capacitive effects,^9,21−23^ limiting their use at low light illumination intensities. Additionally, at higher intensities such as those characteristic of solar fluxes, CE experiments on thin-film diodes based on low-mobility materials (such as OPVs) may be inadvertently affected by both incomplete CE and limitations set by the RC time; how these effects influence the determination of β and δ is not known.

In this work, we investigate the validity of using transient CE experiments to probe the carrier density-dependence of the charge carrier recombination rate in OPVs. Specifically, we clarify the role of mobility and RC time in the evaluation of β and δ. Indeed, utilizing an open-source, transient drift-diffusion model presented in the Supporting Information,^21,24−27^ we find that incomplete CE leads to mobility-dependent and series resistance-dependent bimolecular recombination rates–artifacts that could be falsely interpreted as evidence for higher-order recombination processes. To overcome these limitations, we provide an alternative framework through which CE measurements may be interpreted, before presenting an analytical model for the recombination coefficient obtained through a CE experiment that accounts for incomplete charge transport. We then validate this model against data simulated using the drift-diffusion model, before demonstrating how the model can be used to correct the carrier density-dependence observed in experimental data.

A schematic diagram of the transient CE experiment is illustrated in Figure 1.^21,28^ Therein, an OPV device with active layer thickness *d*, electrical cross-sectional area *A*, shunt resistance *R*_sh_, and series resistance *R*_s_ is connected in series with (i) a load resistance *R*_L_ and (ii) a voltage source that, at a given time *t*, applies voltage *V*_app_(*t*) to the circuit. The current density *J*(*t*) is subsequently obtained by measuring the voltage drop *V*_*L*_(*t*) across the load using an oscilloscope (*V*_*L*_(*t*) = *J*(*t*)*AR*_L_). Ideally, *R*_L_ and *R*_s_ are both assumed to be small enough to not significantly influence the voltage drop *V*_drop_(*t*) across the device during the experiment. Here,
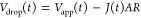
1where *R* = *R*_s_ + *R*_L_ is the total series resistance of the external circuit. Using this setup, a CE experiment proceeds in two stages.

**Figure 1:**
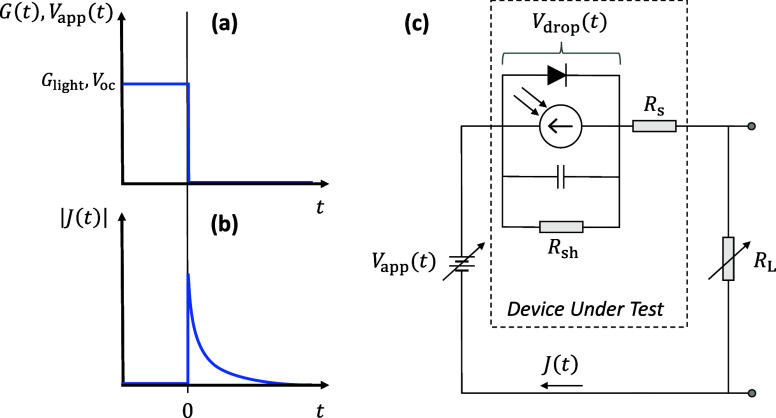
Schematic of the transient CE experiment. (a) As a function of time (*t*), the generation rate (*G* ≈ *G*_light_) across the active layer of the OPV device (proportional to the intensity of the light) and the voltage applied to the circuit (*V*_app_). The device is kept at open-circuit conditions under steady-state illumination until time *t* = 0, when the light and voltage sources are turned off simultaneously, leading to (b) the extraction of a current density (*J*) from the device, from which an extracted carrier density may be determined. (c) A circuit diagram illustrating the transient CE experiment, including the equivalent circuit of the OPV device under test (indicated by the dashed box), which is connected in series with a variable voltage source and a variable load resistance.

In the first stage of the CE experiment, the photovoltaic device is kept at open-circuit under steady-state illumination (*V*_app_(*t*) = *V*_drop_(*t*) = *V*_oc_, the open-circuit voltage). Assuming uniform carrier distributions (i.e., sufficiently high light intensities), the charge carrier recombination rate  under these conditions relates to the corresponding (open-circuit) carrier density (*n*_oc_) within the device via
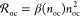
2where β may depend on the carrier density. Under these circumstances, the charge carrier recombination rate is perfectly balanced with the (spatially averaged) generation rate (*G*_light_): . Hence, for a given light intensity,  may be inferred from the associated generation rate *G*_light_.

In the second stage of the CE experiment, which commences at time *t* = 0, the light incident on the device and the voltage applied to the circuit are turned off simultaneously, while the corresponding induced current response *J*(*t*) is measured. The current is given by the sum of the spatially averaged conduction (*J*_c_) and displacement (*J*_d_) current densities across the device: *J*(*t*) = *J*_c_(*t*) + *J*_d_(*t*). By integrating the resulting *J*_c_(*t*), the extracted carrier density (*n*_CE_) may then be evaluated through
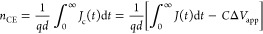
3where the right-hand term accounts for the extracted carrier density associated with the displacement current induced by the change in applied voltage (Δ*V*_app_), with  being the geometric capacitance of the device (ϵ_r_ and ϵ_0_ are the relative permittivity of the OPV material and the vacuum permittivity, respectively). Note that a reverse bias may also be applied at this stage to hasten CE; this method is referred to as bias-assisted charge extraction (BACE).^28^ For simplicity, however, we assume that *V*_app_(*t* > 0) = 0, and thus Δ*V*_app_ = *V*_oc_. Note that the conclusions made in the following discussion can be, and indeed are, extended to BACE measurements.

In the ideal case that *n*_CE_ = *n*_oc_, the CE experiment may be used to directly evaluate *n*_oc_ at any given light intensity. The recombination rate constant β can then be determined by measuring *n*_CE_ for a variety of initial light intensities (i.e., varied *G*_light_) and assuming the following relationship
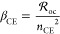
4while the reaction order is estimated from the slope: . Here, β_CE_ denotes the recombination rate constant obtained from the CE experiment, whereas the steady-state recombination rate, , can either be estimated from the corresponding *G*_light_ (noting that ), e.g., via the saturated photocurrent density *J*_gen_ = *qG*_light_*d* measured in reverse-bias under steady-state conditions; or in conjunction with a transient photovoltage (TPV) measurement.^21^Equation 4 allows for the detection of any carrier density dependence of β_CE_ (i.e., deviation from bimolecular recombination), along with the associated recombination order. However, Equation 4 relies on the assumptions of uniform carrier distributions (prior to *t* = 0) and complete CE (after *t* = 0). The former is generally violated at low intensities by the manifestation of a capacitance-limited regime.^21−23^ The validity of the latter is expected to depend on the electron and hole mobility (μ_*n*_ and μ_*p*_, respectively). For materials with low carrier mobilities (like OPVs), the time it takes to extract all excess carriers may, depending on the carrier density, be longer than the prevailing recombination lifetime; under such conditions, a substantial number of carriers will recombine during the extraction process, inevitably resulting in a violation of the underpinning assumption that *n*_CE_ = *n*_oc_.

To clarify the influence of carrier mobility on the extracted carrier density, several light intensity-dependent (i.e., carrier density-dependent) CE experiments were simulated using a numerical drift-diffusion model. The results for the case of negligible series resistance (*R* = *R*_s_ + *R*_L_ = 0), simulated using the parameters summarized in Table S1 in the Supporting Information, are illustrated in Figure 2a. Therein, the input recombination coefficient was fixed at β = 1.20 × 10^–11^ cm^3^ s^–1^, corresponding to the case of a purely bimolecular recombination rate (δ = 2). For all results presented in this work, Ohmic contacts were assumed, such that the effects of surface recombination are negligible. From Figure 2a, it is evident that *n*_CE_ depends on the mobility, especially at higher intensities where lower mobilities were found to correlate with a reduced *n*_CE_, consistent with incomplete CE. The resultant bimolecular recombination rate constant determined using Equation 4 is plotted in Figure 2b, and the corresponding recombination order δ_CE_ is plotted against the recombination rate and the extracted carrier density in Figure 2c and 2d, respectively. From Figure 2, it can be seen that the obtained β_CE_ increases with *n*_CE_, despite the fact that the input β (shown by the dashed line) was fixed. This, in turn, leads to an overestimation of the recombination order that increases with reduced mobility.

**Figure 2:**
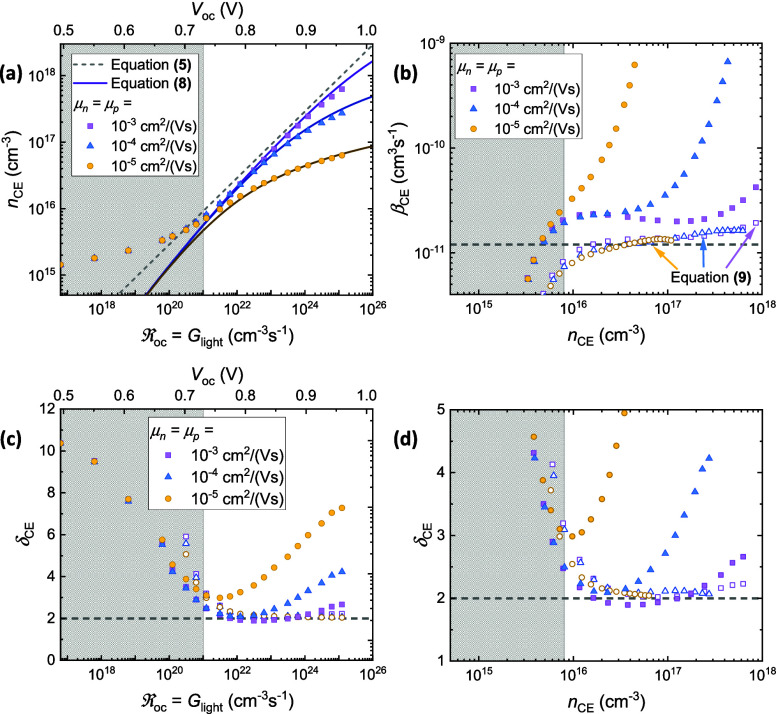
CE measurements simulated in the case of negligible series resistance for a variety of charge carrier mobilities (with μ_*n*_ = μ_*p*_). (a) The extracted carrier density (*n*_CE_), plotted as a function of the open-circuit recombination rate . Here, the dashed line indicates the *n*_CE_ expected from eq 5 in the ideal case that *n*_CE_ = *n*_oc_, while the symbols indicate the carrier densities determined by applying eq 3 to simulated current transients. The solid lines indicate the extracted carrier density in the analytical model given by eq 8. For reference, the open-circuit voltage is included as an upper axis, where *qV*_oc_ ∝ *k*_B_*T* ln(*G*_light_). (b) The bimolecular recombination rate constants determined using eq 4 and eq 9 are indicated by filled-in and empty symbols, respectively. The dashed gray line indicates the simulation input value, β = 1.20 × 10^–11^ cm^3^ s^–1^. The corresponding reaction order δ_CE_ is plotted as a function of the recombination rate and the extracted carrier density in (c) and (d), respectively. In all panels, the gray shaded regions at low light intensities/open-circuit voltages indicate approximately where capacitive effects begin to limit the experiment.^21^

To verify that the apparent variation in β_CE_ with increasing carrier density is indeed an artifact of incomplete CE—and to write a corrected form of eq 4—we next present a simple-yet-accurate analytical model. To simplify the treatment, we assume that the photoactive layer is sufficiently thin, and *G*_light_ intense enough, for the photogenerated electron and hole density profiles to be essentially flat across the entire active layer in the steady state prior to CE, such that capacitive effects can be neglected.^21^ Under these assumptions, the carrier densities of electrons (*n*) and holes (*p*) can initially (i.e., at *t* ≤ 0) be approximated (at all positions *x*) as equal to *n*_oc_. In accordance with eq 2, we expect
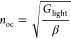
5for purely bimolecular recombination.

Immediately after the light and applied voltage have been turned off (*t* > 0), the sudden change in biasing conditions causes the photogenerated carriers to start drifting under the influence of the electric field induced within the device. In the case of negligible series resistance, the corresponding magnitude of the electric field (*F*) may be approximated as *F* ≈ |Δ*V*_app_|/*d* = *V*_oc_/*d*. Neglecting diffusive effects, electrons are extracted at *x* = *d*, moving out of the active layer in a uniform sheet with speed μ_*n*_*F*, leaving an electron-depleted region of width *l*_*n*_(*t*) = μ_*n*_*Ft* in their wake. At the same time, holes drift as a uniform sheet with velocity μ_*p*_*F* in the opposite direction, moving out of the active layer at the contact at *x* = 0 while leaving a hole-depleted region of width *l*_*p*_(*t*) = μ_*p*_*Ft* behind. Thus, the carrier densities can be approximated as *n*(*x*,*t*) ≈ *n*(*t*) for *x* ≥ *l*_*n*_(*t*), and *n*(*x*,*t*) ≈ 0 elsewhere; while *p*(*x*,*t*) ≈ *p*(*t*) for *x* ≤ *d* – *l*_*p*_(*t*), and *p*(*x*,*t*) ≈ 0 elsewhere. During this extraction process, however, recombination between electrons and holes will occur simultaneously within the overlapping region *l*_*n*_(*t*) ≤ *x* ≤ *d* – *l*_*p*_(*t*) for *t* ≤ *t*_tr_, where
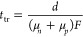
6

After this transit time (when *t* > *t*_tr_), the excess electron and hole profiles have no overlap within the active layer, and recombination between them ceases.

Since uniform carrier distributions are assumed at *t* = 0, in the absence of recombination the extracted charge carrier density becomes *n*_CE_ = *n*_oc_ in this case. For ohmic contacts, however, there is generally a considerable energy level bending near the contact, suppressing the electron (hole) density near the anode (cathode) in this region. The width of this region is approximately given by the associated Debye screening length .^29^ We found that this effect can be accounted for by effectively replacing *n*_oc_ with  for small *L*_D_.

The effect of bimolecular recombination during the extraction process can be shown to reduce the extracted carrier density as ,^15,21^ where the spatially averaged recombination rate is given by . On the other hand, as bimolecular recombination diminishes *n*(*t*) and *p*(*t*) through , with *n*(0) = *p*(0) = *n*_oc_, this leads to a time-dependent carrier density of the form
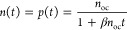
7where  corresponds to the carrier lifetime associated with the bimolecular recombination process. Hence, the extracted carrier density may be written as (see section S1 of the Supporting Information):
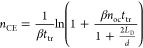
8where β*n*_oc_*t*_tr_ is the ratio between the transit time and the carrier lifetime, which depends on the intensity via *n*_oc_ through eq 5. Note that, within the logarithm, we have corrected *n*_oc_ for the Debye screening length to (partially) account for the effect of Ohmic contacts. Accordingly, only when *t*_tr_ is much shorter than the lifetime associated with bimolecular recombination (β*n*_oc_*t*_tr_ ≪ 1) is there complete CE (*n*_CE_ → *n*_oc_^′^). However, for low mobilities and/or high light intensities (β*n*_oc_*t*_tr_ > 1), charge is lost due to recombination during the transient extraction process, leading to *n*_CE_ < *n*_oc_. As shown by the close agreement between the drift-diffusion data and the solid lines in Figure 2a, eq 8 reproduces the effect of incomplete CE well.

Using the formula for the corrected *n*_CE_ given in eq 8, an amended form of the expression for the bimolecular recombination rate constant given in eq 4 can be written as
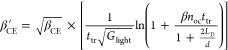
9

Using eq 9, the extracted bimolecular recombination rate constants previously plotted as solid symbols in Figure 2b were corrected, with the amended values plotted in the same panel as empty symbols. The corrected reaction orders are plotted against  and *n*_CE_ in Figure 2c and 2d, respectively. Indeed, for all mobilities the obtained β_CE_^′^ values are within a factor of 2 from the simulation input value (β = 1.2 × 10^–11^ cm^3^ s^–1^), giving a reaction order δ_CE_ ≈ 2 for most light intensities. Compared with the orders-of-magnitude fluctuations and apparent *n*_CE_-dependence that was observed in β_CE_ for the uncorrected case, it is clear that incomplete CE can lead to erroneous evaluations of the bimolecular recombination rate constant. However, such variations are not an intrinsic limitation of the CE experiment. The fault instead lies with the analysis and the *n*_CE_ = *n*_oc_ assumption that underpins eq 4. While the β_CE_^′^ values determined using eq 9 are far more accurate than the β_CE_ values determined using eq 4, there are still fluctuations in the corrected data points; these are likely due to additionally complexities, such as inhomogeneities in the carrier density profiles,^16,21^ which are neglected in the analytical model.

A similar incomplete CE phenomenon may be observed when accounting for the resistance–capacitance (RC) effects that arise from the combined series resistance of the circuit (*R* = *R*_s_ + *R*_L_). As demonstrated by the simulations shown in Figure 3a, which were simulated using device area *A* = 0.1 cm^2^, increasing the series resistance leads to reduced CE at higher light intensities. This, in turn, leads to an apparent increase in the bimolecular recombination rate constant, as shown in Figure 3b. Ultimately, this culminates in an overestimation of the reaction order, as illustrated by the curves in Figure 3c and 3d. The influence of RC effects is explored further for varied *A* in Figure S1 of the Supporting Information. Primarily, this effect can be attributed to resistive voltage losses induced by the large extraction current (present at high intensities), reducing the electric field across the device as *F*(*t*) ≈ |Δ*V*_app_ – *ARJ*(*t*)|/*d* = |*V*_oc_ – *ARJ*(*t*)|/*d*. After accounting for resistive losses dominate, such that the RC time τ_RC_ = *ARC* ≠ 0, we find that the extracted carrier density and corrected bimolecular recombination rate constant, denoted as *n*_CE_^RC^ and β_CE_^RC^, respectively, can be analytically approximated with (see section S2 of the Supporting Information)
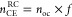
10a
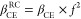
10b
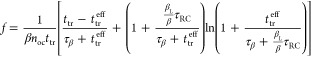
10cwhere *f* is a correction factor,  is the recombination lifetime (including the Debye screening factor), β_*L*_ = *q*(μ_*n*_ + μ_*p*_)/ϵ_r_ϵ_0_ is the Langevin recombination rate constant, and *t*_tr_^eff^ is the effective transit time, which can be approximated (see Supporting Information**)** as
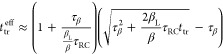
11

**Figure 3:**
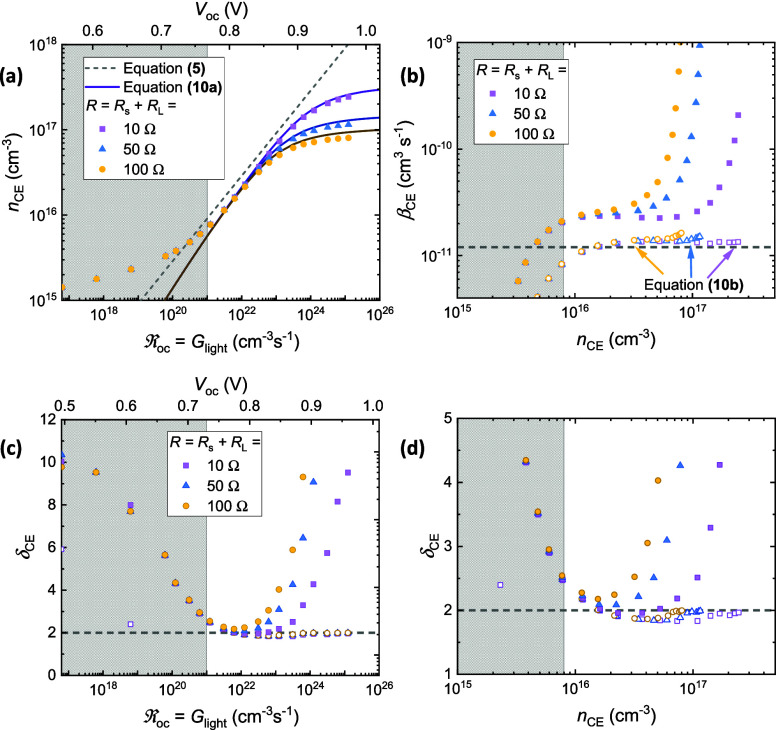
Simulated CE measurements for a device with area *A* = 0.1 cm^2^, where the combined series resistances of the circuit (*R* = *R*_s_ + *R*_L_) has been varied while the mobility was fixed at μ = 10^–3^cm^2^/(Vs). (a) The extracted carrier density (*n*_CE_), plotted as a function of the open-circuit recombination rate . Here, the symbols indicate *n*_CE_ determined by applying eq 3 to the simulated current transients, the dotted line indicates eq 5, and the solid lines indicate eq 10a. (b) The corresponding bimolecular recombination rate constants determined using eq 4 and 10b are illustrated by the filled-in symbols and empty symbols, respectively. The dashed gray line indicates the simulation input value, β = 1.20 × 10^–11^ cm^3^ s^–1^. The resultant recombination order, determined using , is plotted as a function of the open-circuit recombination rate and the extracted carrier density in (c) and (d), respectively. In all panels, the gray shaded regions at low light intensities/open-circuit voltages illustrate where capacitive effects begin to limit the experiment.^21^

As expected, in the limit that *R* → 0, the analytical expressions for the extracted carrier density and the bimolecular recombination rate constant given in eq 10 reduce to eqs 8 and 9, respectively. Eq 10 was used to simulate the solid lines in Figure 3a, as well as the empty data points in Figure 3b. From this figure, one can see that accounting for the combined series resistance of the external circuit is a necessity to accurately determine β.

Finally, to demonstrate how resistances can lead to incomplete CE and possibly an apparent carrier density-dependence in the obtained bimolecular recombination rate constant, we applied the analytical model described by eq 10 to BACE measurements made by Hosseini et al. to correct the experimentally determined bimolecular recombination rate constants of two OPV donor:acceptor blends. Both blends used a naphtho[1,2-c:5,6-c′]bis([1,2,5]thiadiazole)-based polymer (NT812) as the donor.^28,30^ While one blend used ITIC as the acceptor, the other used PC_70_BM (for chemical definitions, see Table S2 in the Supporting Information).^28^ Note that a nonzero voltage bias was accounted for in the following calculations (see section S2 of the Supporting Information). By developing and applying an open-source computational tool that employs a least-squares method to estimate β (freely available online; see Supporting Information),^31^ we found the carrier density-dependence observed in the β_CE_ data plotted in Figure 4 can be corrected for if we effectively assume *R* = 1000 Ω, suggesting that such variations could be described by incomplete CE, rather than higher-order (δ > 2) recombination processes. The trend in β_CE_^′^ versus *n*_CE_ observed in Figure 4 now reflects that seen in Figures 2b and 3b. In particular, the large increase in measured β_CE_ has been tempered with a smaller rise followed by a fall. Note that the parameters used to correct this data are provided in Table S3 in the Supporting Information, alongside an expression for the resistance-dominated voltage at *t* ≪ τ_RC_.

**Figure 4:**
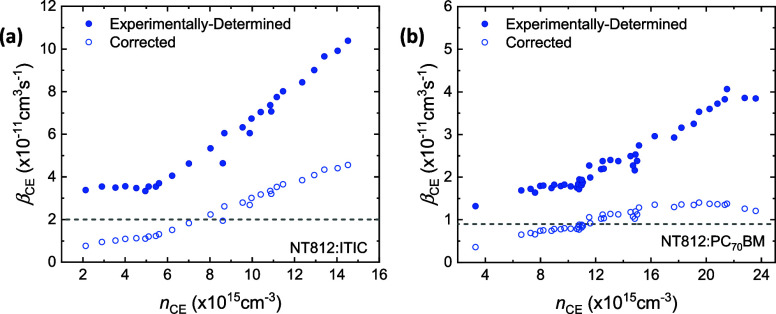
Using the analytical model for CE to correct experimentally determined bimolecular recombination rate constant measurements of an OPV device with (a) an NT812:ITIC active layer and (b) an NT812:PCBM active layer. Here, the filled-in data points represent the experimentally determined bimolecular recombination rate constant, determined as a function of the carrier density using BACE measurements (where *V*_app_(*t* > 0) ≠ 0) by Hosseini et al.^28^ The empty data points indicate the corresponding corrected bimolecular recombination rate constants.

In conclusion, we have developed a simple analytical model to describe CE experiments. By applying this model to drift-diffusion and experimental data, we have shown that bimolecular recombination of excess charge carriers during the transient extraction process of a CE experiment generally leads to incomplete CE in diode devices such as OPVs with low carrier mobilities. This, in turn, may result in an erroneous evaluation of the bimolecular recombination rate constant and an overestimation of the recombination order in low-mobility diodes. To overcome these underlying limitations of the CE experiment, we have presented a new framework for analysis as well as a computational tool^31^ for correcting the bimolecular recombination rate constant obtained from experimentally determined CE measurements. We have demonstrated how this model can be applied by correcting the carrier density-dependence of published bimolecular recombination rate data for OPV devices, demonstrating that the technique and computational tool can be used to reinterpret existing measurements in the literature.

## Data Availability

The data that support the results of this work will be made available upon request from the corresponding authors.

## Supporting Information Available

Additional simulations, derivations, and descriptions of parameter extraction; a complete description of the drift-diffusion model and an accompanying, open-source computational tool with an accessible graphical user interface. (PDF)
Transparent Peer Review report available (PDF)
