# Supplementary material for: A New Framework for Understanding Recombination-Limited Charge Extraction in Disordered Semiconductors
Source: J Phys Chem Lett. 2024 Apr 16;15(16):4416–21. doi: 10.1021/acs.jpclett.4c00218 (PMC11057038; doi:10.1021/acs.jpclett.4c00218)
Supplement: Supplementary file 1 — jz4c00218_si_001.pdf [file jz4c00218_si_001.pdf]

Supporting Information

A New Framework for Understanding Recombination-Limited Charge Extraction in Disordered Semiconductors

Austin M. Kay[*1], Drew B. Riley*[*1], Paul Meredith*[1], Ardalan Armin*[1], Oskar J. Sandberg*[*2]

[1]Sustainable Advanced Materials (Sêr-SAM), Centre for Integrative Semiconductor Materials (CISM), Department of Physics, Swansea University Bay Campus, Swansea SA1 8EN, United Kingdom

[2]Physics, Faculty of Science and Engineering, Åbo Akademi University, 20500 Turku, Finland

Email: a.m.kay.954708@swansea.ac.uk; d.b.riley@swansea.ac.uk; oskar.sandberg@abo.fi

Table of Contents

Part I. Theoretical Modelling of the Charge Extraction Experiment 3

S1. Analytical Model for the Charge Extraction Experiment 4

S2. Bimolecular Recombination Rate Constant Correction 9

Part II. The Drift-Diffusion Model 12

S3. List of Symbols and Abbreviations 12

S4. Energetic Landscape 17

S5. The System of Differential Equations 22

S6. Generation and Recombination 27

S7. Boundary Conditions 29

S8. Normalisation 31

S9. Discretising the Drift-Diffusion Equations 33

S10. Discretising the Voltage Drop (Including Resistance-Capacitance Effects) 40

S11. Iteration and Convergence 42

Part I. Theoretical Modelling of the Charge Extraction Experiment

In this part of the Supporting Information, the analytical model for the charge extraction (CE) experiment is derived. Before this, a summary of the parameters used to simulate mobility and series-resistance dependent CE experiments, as discussed in the main text, is provided in **Table S1**.

Table S1: The parameters input into the drift-diffusion model described in **Part II** to simulate transient CE experiments in low-mobility semiconductors.

Parameter	Symbol	Value
Active layer cross-sectional area	A	$[0.01, 0.10, 1.00]$ cm^2
Active layer thickness	d	100 nm
Temperature	T	293.15 K
Energetic gap	E_{g}	1.2 eV
Electron and hole mobilities	μ_n, μ_p	$[10^{-3}, 10^{-4}, 10^{-5}]$ cm^{2}V^{-1}s^{-1}
Langevin recombination reduction factor	γ	0.1
Effective electron and hole densities	n_{c}, n_{v}	10^{20} cm^{-3}
Relative permittivity	ϵ_{r}	3
One-Sun generation rate	G_{light}	6.24×10^{21} cm^{-3}s^{-1}
Cathode electron injection barrier	Φ_n^{cat}	0
Anode hole injection barrier	Φ_p^{an}	0
Series resistance	R_{s}	[0, 10, 50, 100] Ω
Shunt resistance	R_{sh}	∞
Load resistance	R_{L}	0

S1. Analytical Model for the Charge Extraction Experiment

To account for bimolecular recombination of excess charge carriers during a transient charge extraction (CE) experiment, an analytical model was derived. In this model, the light intensity is assumed sufficiently intense that capacitive effects may be ignored and that the charge carrier profiles are mostly uniform across the active layer. Treating the transient extraction process as the motion of sheets of charge then, neglecting diffusive effects, the electron density (n) at time t may be approximated with

$$n(x,t) = \begin{cases} n(t), & \text{for} \quad x \geq l_n(t). \\ 0, & \text{otherwise.} \end{cases} \quad \textbf{(S1.1)}$$

Here, $l_n(t)$ is the drift distance the electrons have propagated towards the cathode at time t. In a similar vein, the hole density (p) at time t may be approximated with

$$p(x,t) = \begin{cases} p(t), & \text{for} \quad x \leq d - l_p(t), \\ 0, & \text{otherwise,} \end{cases} \quad \textbf{(S1.2)}$$

where $l_p(t)$ is the corresponding drift distance propagated towards the anode by the sheet of holes. Consequently, recombination between electrons and holes occurs only in the region $l_n(t) \leq x \leq d - l_p(t)$ up until the time t_{tr}, defined by $l_n(t_{\mathrm{tr}}) \equiv d - l_p(t_{\mathrm{tr}})$. In this region, bimolecular recombination between carriers obeys $\frac{\partial n(t)}{\partial t} = \frac{\partial p(t)}{\partial t} = -\beta n(t)p(t)$, where β is the bimolecular recombination rate constant. At time $t = 0$, it is assumed electrons and holes have the same density defined by the open-circuit conditions: $n(0) = p(0) = n'_{\mathrm{oc}} = n_{\mathrm{oc}}/(1 + \frac{2L_{\mathrm{D}}}{d})$, where $n_{\mathrm{oc}} = \sqrt{\frac{G_{\mathrm{light}}}{\beta}}$ is the open-circuit carrier density and $L_{\mathrm{D}} = \sqrt{2\epsilon_{\mathrm{r}}\epsilon_0 k_{\mathrm{B}}T/q^2 n_{\mathrm{oc}}}$ is the Debye screening length (ϵ_{r} and ϵ_0 are the relative and vacuum permittivity, respectively, while k_{B}, T, and q have their usual definitions as the Boltzmann constant, the temperature, and the elementary charge, respectively). Consequently, for times $t < t_{\mathrm{tr}}$, the time-dependent carrier densities in the region $l_n(t) \leq x \leq d - l_p(t)$ are given by

$$n(t) = p(t) = \frac{n'_{\mathrm{oc}}}{1 + \beta n'_{\mathrm{oc}} t}, \qquad \textbf{(S1.3)}$$

To determine the carrier density n_{CE} extracted during a CE experiment, the carrier density lost due to this bimolecular recombination must be accounted for. This can be done using[1]

$$n_{\mathrm{CE}} = n'_{\mathrm{oc}} - \int_0^{t_{\mathrm{tr}}} \bar{\mathcal{R}}_{\mathrm{CE}}(t)\,\mathrm{d}t, \qquad \textbf{(S1.4)}$$

where the spatially-averaged recombination rate $\bar{\mathcal{R}}_{\mathrm{CE}}(t)$ is, in this case, given by

$$\bar{\mathcal{R}}_{\mathrm{CE}}(t) = \frac{1}{d}\int_0^d \beta n(x,t) p(x,t)\,\mathrm{d}x = \beta \times \left(\frac{n'_{\mathrm{oc}}}{1 + \beta n'_{\mathrm{oc}} t}\right)^2 \times \left(1 - \frac{[l_n(t) + l_p(t)]}{d}\right). \qquad \textbf{(S1.5)}$$

To progress further, analytical expressions for the width of the depletion regions, $l_n(t)$ and $l_p(t)$, must be determined.

In general, the effect of an external series resistance $R = R_{\mathrm{s}} + R_{\mathrm{L}}$ (where R_{s} is the series resistance of the diode and R_{L} is the load resistance of the external circuit) is to modify the change in voltage across the device (when the bias is switched off) as $\Delta V_{\mathrm{drop}}(t) \approx V_{\mathrm{oc}} - J(t)RA$, depending on the current flow throughout the circuit. Correspondingly, the rate of change of the depletion width for electrons, induced by the change in voltage, may be approximated as

$$\frac{\mathrm{d}l_n(t)}{\mathrm{d}t} = \frac{\mu_n \Delta V_{\mathrm{drop}}(t)}{d} \approx \frac{\mu_n}{d}[V_{\mathrm{oc}} - J(t)AR]. \qquad \textbf{(S1.6)}$$

An otherwise identical expression applies for the rate of change of the width of the hole depletion region, but with μ_n replaced by μ_p. By first considering the special case of negligible series resistance, Equation **(S1.6)** may be solved to find $l_n(t) = \mu_n F t$, where the electric field across the bulk is given by $F = \frac{|\Delta V_{\mathrm{drop}}|}{d} \approx \frac{V_{\mathrm{oc}}}{d}$. Consequently, with the time that recombination ceases in this low-resistance limit being given by

$$t_{\mathrm{tr}} = \frac{d}{(\mu_n + \mu_p)F}, \qquad \textbf{(S1.7)}$$

the extracted carrier density may be then approximated with

$$n_{\mathrm{CE}} = n'_{\mathrm{oc}} - \beta(n'_{\mathrm{oc}})^2 \int_0^{t_{\mathrm{tr}}} \frac{\left(1 - \frac{t}{t_{\mathrm{tr}}}\right)}{(1 + \beta n'_{\mathrm{oc}} t)^2} \mathrm{d}t = n'_{\mathrm{oc}} \times \frac{\ln(1 + \beta n'_{\mathrm{oc}} t_{\mathrm{tr}})}{\beta n'_{\mathrm{oc}} t_{\mathrm{tr}}}. \quad \textbf{(S1.8)}$$

This expression was used in the negligible series resistance investigation presented in **Figure 2** of the main text. In general, however, the series resistance is not negligible. For moderate RC times, and high enough extraction currents, the total current $J(t)$ induced by extraction may, in this case, be approximated by

$$J(t) \approx qn(t)\left[\frac{\mathrm{d}l_n(t)}{\mathrm{d}t} + \frac{\mathrm{d}l_p(t)}{\mathrm{d}t}\right]. \quad \textbf{(S1.9)}$$

where $n(t)$ was defined in Equation **(S1.3)**. Noting that $\frac{1}{\mu_n}\frac{\mathrm{d}l_n(t)}{\mathrm{d}t} = \frac{1}{\mu_p}\frac{\mathrm{d}l_p(t)}{\mathrm{d}t}$, the extracted current density can then be expressed in terms of the rate of change in the electron depletion region width via

$$J(t) = qn(t)\left(\frac{\mu_n + \mu_p}{\mu_n}\right)\frac{\mathrm{d}l_n(t)}{\mathrm{d}t}. \quad \textbf{(S1.10)}$$

Combining Equation **(S1.10)** with Equation **(S1.6)** gives

$$\frac{\mathrm{d}l_n(t)}{\mathrm{d}t} = \frac{\mu_n V_{\mathrm{oc}}}{d\left(1 + \frac{ARC_0 \beta_{\mathrm{L}} n'_{\mathrm{oc}}}{(1 + \beta n'_{\mathrm{oc}} t)}\right)}. \quad \textbf{(S1.11)}$$

where the Langevin recombination rate constant is defined by $\beta_{\mathrm{L}} = q[\mu_n + \mu_p]/\epsilon_{\mathrm{r}}\epsilon_0$, while the geometric capacitance is given by $C_0 = \frac{\epsilon_{\mathrm{r}}\epsilon_0}{d}$. Equation **(S1.11)** can be readily solved for $l_n(t)$, while an analogous expression for holes can be found using $\frac{1}{\mu_n}\frac{\mathrm{d}l_n(t)}{\mathrm{d}t} = \frac{1}{\mu_p}\frac{\mathrm{d}l_p(t)}{\mathrm{d}t}$. After combining these together, the following expression can be obtained for the sum of the normalised depletion widths:

$$\frac{l_n(t) + l_p(t)}{d} = \frac{t}{t_{\mathrm{tr}}} - \frac{\beta_{\mathrm{L}}}{\beta}\frac{\tau_{\mathrm{RC}}}{t_{\mathrm{tr}}} \ln\left[1 + \frac{t}{\tau_\beta + \frac{\beta_{\mathrm{L}}}{\beta}\tau_{\mathrm{RC}}}\right]. \quad \textbf{(S1.12)}$$

where $\tau_{\mathrm{RC}} = ARC_0$ is the RC time constant and $\tau_\beta = \frac{1}{\beta n'_{\mathrm{oc}}}$ is the lifetime associated with the recombination process. We note that Equation **(S1.12)** reduces to the expected expression in the limit that $R \to 0$

(corresponding to $\tau_{\mathrm{RC}} \to 0$). The effective transit time ($t_{\mathrm{tr}}^{\mathrm{eff}}$) at which recombination ceases (defined by $l_n(t_{\mathrm{tr}}^{\mathrm{eff}}) + l_p(t_{\mathrm{tr}}^{\mathrm{eff}}) \equiv d$) can be determined numerically as the solution of

$$t_{\mathrm{tr}}^{\mathrm{eff}} - \frac{\beta_{\mathrm{L}}}{\beta}\tau_{\mathrm{RC}} \ln\left[1 + \frac{t_{\mathrm{tr}}^{\mathrm{eff}}}{\tau_\beta + \frac{\beta_{\mathrm{L}}}{\beta}\tau_{\mathrm{RC}}}\right] - t_{\mathrm{tr}} = 0, \tag{S1.13}$$

noting that t_{tr} is defined by Equation **(S1.7)** as the corresponding time in the low-resistance case. Using a second-order Taylor expansion of the natural logarithm, an approximation for $t_{\mathrm{tr}}^{\mathrm{eff}}$ may be written as

$$t_{\mathrm{tr}}^{\mathrm{eff}} \approx \left(1 + \frac{\tau_\beta}{\tau_{\mathrm{RC}}'}\right)\left(-\tau_\beta + \sqrt{\tau_\beta^2 + 2\tau_{\mathrm{RC}}' t_{\mathrm{tr}}}\right), \tag{S1.14}$$

where $\tau_{\mathrm{RC}}' = \frac{\beta_{\mathrm{L}}}{\beta}\tau_{\mathrm{RC}}$ has been defined to simplify notation. Using the effective transit time, the extracted charge carrier density can be calculated using

$$\begin{aligned} \frac{n_{\mathrm{CE}}}{n_{\mathrm{oc}}'} &= 1 - \frac{1}{n_{\mathrm{oc}}'}\int_0^{t_{\mathrm{tr}}^{\mathrm{eff}}} \bar{\mathcal{R}}_{\mathrm{CE}}(t)\,\mathrm{d}t \\ &= 1 - \frac{\tau_\beta}{t_{\mathrm{tr}}}\int_0^{t_{\mathrm{tr}}^{\mathrm{eff}}} \frac{1}{(\tau_\beta + t)^2}\left(t_{\mathrm{tr}} - t + \tau_{\mathrm{RC}}' \ln\left[1 + \frac{t}{\tau_\beta + \tau_{\mathrm{RC}}'}\right]\right)\mathrm{d}t. \end{aligned} \tag{S1.15}$$

After evaluating the integral, we find that the ratio of the extracted carrier density to the open-circuit carrier density can be written as

$$\frac{n_{\mathrm{CE}}}{n_{\mathrm{oc}}'} = \frac{\tau_\beta}{t_{\mathrm{tr}}}\left[\left(\frac{t_{\mathrm{tr}} - t_{\mathrm{tr}}^{\mathrm{eff}}}{t_{\mathrm{tr}}^{\mathrm{eff}} + \tau_\beta}\right) + \left(1 + \frac{\tau_{\mathrm{RC}}'}{t_{\mathrm{tr}}^{\mathrm{eff}} + \tau_\beta}\right)\ln\left(1 + \frac{t_{\mathrm{tr}}^{\mathrm{eff}}}{\tau_\beta + \tau_{\mathrm{RC}}'}\right)\right], \tag{S1.16}$$

which reduces to Equation **(S1.8)** in the limit that $R \to 0$ (as $\tau_{\mathrm{RC}} \to 0$ and $t_{\mathrm{tr}}^{\mathrm{eff}} \to t_{\mathrm{tr}}$), as expected.

To explore the effect of the device area A on the bimolecular recombination rate determined using the charge extraction experiment, the drift-diffusion model described in **Part II** was used to simulate the curves shown in **Figure S1**. Therein the extracted carrier density is plotted against the open-circuit generation rate (G_{light}) for three different device areas in Figure S1a-c for three resistances each. The corresponding bimolecular recombination rate constants determined using $\beta_{\mathrm{CE}} = \frac{G_{\mathrm{light}}}{n_{\mathrm{CE}}^2}$, and the corrected

rate constants determined using $\beta'_{\mathrm{CE}} = \beta_{\mathrm{CE}} \times \frac{n_{\mathrm{CE}}^2}{n_{\mathrm{oc}}^2}$, are plotted in Figure S1d-f. From this figure, it is clear that an increase in the device area results in increased RC effects, which further limit the charge extraction experiment. Using the analytical model given by Equation **(S1.16)**, however, these limitations can be overcome to correct β_{CE}.

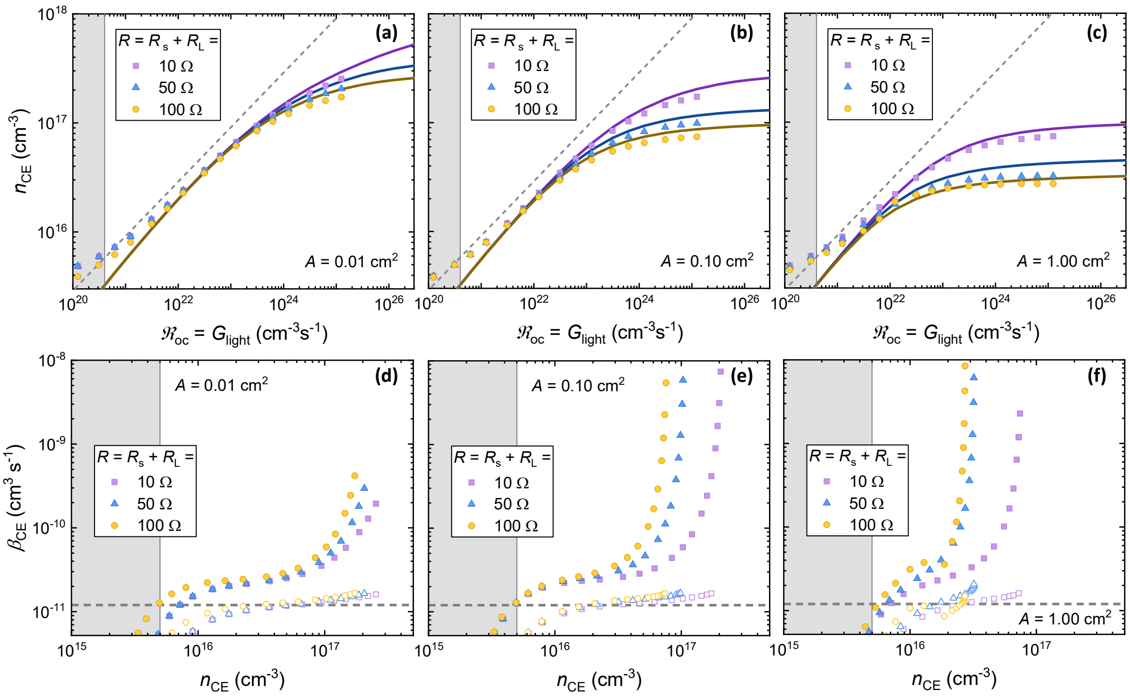

Figure S1: Correcting the charge extraction experiment for a variety of different cross-sectional areas (A) and combined series resistances ($R = R_{\mathrm{s}} + R_{\mathrm{L}}$). In **(a-c)**, the extracted carrier density (n_{CE}) is plotted against the open-circuit recombination/generation rate ($\mathcal{R}_{\mathrm{oc}} = \beta n_{\mathrm{oc}}^2 \approx G_{\mathrm{light}}$) for devices with area $A =$ 0.01, 0.10, and 1.00 cm^2, respectively. Therein, the dashed lines indicate the $n_{\mathrm{CE}} = n_{\mathrm{oc}}$ limit, while the solid lines indicate Equation **(S1.16)**. The corresponding bimolecular recombination rate constants determined using $\beta_{\mathrm{CE}} = \frac{G_{\mathrm{light}}}{n_{\mathrm{CE}}^2}$ are indicated by the solid symbols in **(d-f)**, while the corrected recombination rate constants determined using Equation **(S1.16)** are indicated by the empty symbols.

S2. Bimolecular Recombination Rate Constant Correction

To correct experimentally-determined bimolecular recombination rate constants using the analytical model described in **Section S1**, a computational tool has been developed and is freely available online.[2] As outlined in the previous section, the carrier density extracted in a CE experiment not only depends on device parameters such as the active layer thickness (d), the cross-sectional area (A), and the total series resistance of the external circuit ($R = R_\mathrm{s} + R_\mathrm{L}$), but also material parameters like the charge carrier mobilities (μ_n and μ_p), the generation rate prior to charge extraction (G_light), and the bimolecular recombination rate constant (β). Correcting experimentally-determined bimolecular recombination rate constants therefore requires knowledge of the transit time (t_tr) at which recombination ends in the $R = 0$ limit, which may be calculated using Equation **(S1.7)**. To this end, the change in voltage drop across the device (ΔV_drop) may be estimated using

$$\Delta V_\mathrm{drop}(t) = (V_\mathrm{oc} + V_\mathrm{bias})\left[1 - \exp\left(-\frac{t}{\tau_\mathrm{RC}}\right)\right] - J(t)AR, \qquad \textbf{(S2.1)}$$

where the content of the square brackets accounts for the RC effect of the external circuit, V_bias is any additional voltage bias applied in a bias-assisted CE (BACE) experiment, and the open-circuit voltage at a given G_light may be determined from the open-circuit voltage and generation rate under one-Sun conditions (denoted as $V_\mathrm{oc}^\odot$ and $G_\mathrm{light}^\odot$, respectively) using

$$V_\mathrm{oc} = V_\mathrm{oc}^\odot + \frac{k_\mathrm{B}T}{q}\ln\left(\frac{G_\mathrm{light}}{G_\mathrm{light}^\odot}\right). \qquad \textbf{(S2.2)}$$

Making use of Equation **(S1.10)**, Equation **(S2.1)** can be rewritten as

$$\Delta V_\mathrm{drop}(t) = \frac{(V_\mathrm{oc} + V_\mathrm{bias})\left[1 - \exp\left(-\frac{t}{\tau_\mathrm{RC}}\right)\right]}{1 + \beta_\mathrm{L}\tau_\mathrm{RC}n(t)}. \qquad \textbf{(S2.3)}$$

For $t \gg \tau_\mathrm{RC}$ the voltage drop could be approximated with

$$\Delta V_{\mathrm{drop}} = \frac{(V_{\mathrm{oc}} + V_{\mathrm{bias}})}{1 + \beta_{\mathrm{L}} \tau_{\mathrm{RC}} n'_{\mathrm{oc}}}. \quad \textbf{(S2.4)}$$

In the case of CE without a bias, $\Delta V_{\mathrm{drop}} = V_{\mathrm{oc}}$. In the case of BACE, however, $\Delta V_{\mathrm{drop}} \rightarrow V_{\mathrm{oc}} + V_{\mathrm{bias}}$. After calculating $t_{\mathrm{tr}} \approx \frac{\mathrm{d}^2}{(\mu_n + \mu_p)\Delta V_{\mathrm{drop}}}$, the effective transit time may be estimated using Equation **(S1.14)**.

To correct the trend observed in the experimentally-determined bimolecular recombination rate constant data presented for NT812:ITIC and NT812:$PC_{70}BM$ in the main text (see **Table S2** for material definitions), Equation **(S2.2)** and Equation **(S2.4)** were employed in conjunction with Equation **(S1.16)** and the parameters presented in **Table S3**.

Table S2: Materials and chemical definitions for the organic photovoltaic blends considered in Figure 4 in the main text.[3]

Material	Chemical Definition
**ITIC**	3,9-bis(2-methylene-(3-(1,1-dicyanomethylene)-indanone))-5,5,11,11-tetrakis(4-hexylphenyl)- dithieno[2,3-d:2′,3′-d′]-s-indaceno[1,2-b:5,6-b′]dithiophene
**$PC_{70}BM$**	[6,6]-phenyl-C70-butyric acid methyl ester

Table S3: Material and device parameters used to correct the trend observed in the bimolecular recombination rate constants of NT812:ITIC and NT812:$PC_{70}BM$ in Figure 4 of the main text.[3]

Parameter	NT812:ITIC	NT812:$PC_{70}BM$	Available or Estimated
d	100 nm	100 nm	Available
A	$0.011\ \mathrm{cm}^2$	$0.011\ \mathrm{cm}^2$	Available
μ_n	$1 \times 10^{-4}\ \mathrm{cm}^2\mathrm{V}^{-1}\mathrm{s}^{-1}$	$4 \times 10^{-4}\ \mathrm{cm}^2\mathrm{V}^{-1}\mathrm{s}^{-1}$	Available
μ_p	$1 \times 10^{-4}\ \mathrm{cm}^2\mathrm{V}^{-1}\mathrm{s}^{-1}$	$2 \times 10^{-3}\ \mathrm{cm}^2\mathrm{V}^{-1}\mathrm{s}^{-1}$	Available
R	$1000\ \Omega$	$1000\ \Omega$	Estimated
V_{bias}	2 V	2 V	Estimated
$V_{\mathrm{oc}}^{\odot}$	0.80 V	0.76 V	Available
$G_{\mathrm{light}}^{\odot}$	$9.6 \times 10^{21}\ \mathrm{cm}^{-3}\mathrm{s}^{-1}$	$8.9 \times 10^{21}\ \mathrm{cm}^{-3}\mathrm{s}^{-1}$	Available
ϵ_{r}	3	3	Estimated

Part II. The Drift-Diffusion Model

In this part of the Supporting Information, the numerical drift-diffusion model used to support this work is presented in its entirety – from Maxwell's equations to Scharfetter and Gummel's iterative approach for numerically solving the continuity equations.[4-6] Additional features, such as the inclusion of resistance-capacitance effects, are also incorporated into the model.[7-9] This model is employed by the drift-diffusion code used to support our work and is freely available on GitHub.[10]

S3. List of Symbols and Abbreviations

Fundamental Constants

ϵ_0	Permittivity of free space
μ_0	Permeability of free space
k_B	The Boltzmann constant
q	Elementary charge

Symbol Definitions

$\vec{\boldsymbol{B}}$	Magnetic field vector
$\vec{\boldsymbol{F}}$	Electric field vector
$\vec{\boldsymbol{J}}$	(Total) Current density vector
$\vec{\boldsymbol{J}}_\mathrm{C}$	Conduction current density vector
$\vec{\boldsymbol{J}}_\mathrm{diff}$	Diffusion current density vector
$\vec{\boldsymbol{J}}_\mathrm{drift}$	Drift current density vector
$\vec{\boldsymbol{J}}_n$	Electron current density vector
$\vec{\boldsymbol{J}}_p$	Hole current density vector
$\vec{\boldsymbol{r}}$	Position vector
β	Bimolecular recombination rate constant

β_{CE}	Bimolecular recombination rate constant determined using a charge extraction experiment
β_{L}	Langevin encounter rate constant
γ	Langevin reduction factor
ΔE_{QFLS}	Quasi-Fermi level splitting (also QFLS)
δ	Width of a sliver of active layer in the discretised model
ϵ_{r}	Relative permittivity
μ_n	Electron mobility
μ_p	Hole mobility
μ_{r}	Relative permeability
ξ	Convergence criterion
ρ	Space-charge density
σ	Electrical conductivity
τ_k	Timestep in a transient simulation
Φ_{an}	Work function of the anode
Φ_{cat}	Work function of the cathode
$\Phi_{n,\mathrm{an}}$	Electron injection barrier at the anode
$\Phi_{n,\mathrm{cat}}$	Electron injection barrier at the cathode
$\Phi_{p,\mathrm{an}}$	Hole injection barrier at the anode
$\Phi_{p,\mathrm{cat}}$	Hole injection barrier at the cathode
χ_{aff}	Electron affinity of a semiconducting material
χ_{IP}	Ionisation potential of a semiconducting material
ψ	Scalar electric potential
$\bar{\psi}$	Electric potential normalisation factor

ψ_0	Arbitrary electric potential at anode-semiconductor (usually zero)
A	(Electrical) Cross-sectional area of a semiconductor device
$\mathcal{B}$	Bernoulli function
C_0	Geometric capacitance
d	Thickness of semiconductor layer in a diode
D_n	Electron diffusion coefficient
D_p	Hole diffusion coefficient
E	Energy of a quantum state
E_c	Minimum energy of the conduction band
E_F	Fermi level of a semiconductor
$E_\mathrm{F,an}$	Fermi level of the anode
$E_\mathrm{F,cat}$	Fermi level of the cathode
$E_{\mathrm{F},n}$	Quasi-Fermi level of electrons in the conduction band/donor HOMOs
$E_{\mathrm{F},p}$	Quasi-Fermi level of holes in the valence band/acceptor LUMOs
E_g	Energy gap of a semiconducting material
E_v	Maximum energy of the valence band
E_vac	Energy of the vacuum level
F	One-dimensional electric field
G	Net generation rate
$\bar{G}$	Generation/recombination rate normalisation factor
G_light	Photoinduced generation rate
J	Total (one-dimensional) current density
$\bar{J}$	Current density normalisation factor
J_C	One-dimensional conduction current density

J_{D}	Spatially-averaged displacement current density
J_n	Electron current density
J_p	Hole current density
l_n	Electron depletion region width
l_p	Hole depletion region width
n	Electron density
$\bar{n}$	Carrier density normalisation factor
n_{an}	Electron density at the anode
n_{c}	Effective density of free electron states
n_{cat}	Electron density at the cathode
n_{CE}	Carrier density extracted in a transient charge extraction measurement
n_{eq}	Equilibrium electron density
n_{int}	Intrinsic carrier density
n_{oc}	Open-circuit carrier density
n_{v}	Effective density of free hole states
p	Hole density
p_{an}	Hole density at the anode
p_{cat}	Hold density at the cathode
p_{eq}	Equilibrium hole density
$\mathcal{R}$	Recombination rate
$\bar{\mathcal{R}}_{\mathrm{CE}}$	Spatially-averaged recombination rate during charge extraction
R	Total series resistance
R_{L}	Load resistance of external circuit
R_{s}	Series resistance of semiconductor device

R_{sh}	Shunt (parallel) resistance of semiconductor device
T	Temperature
t	Time
$\bar{t}$	Time normalisation factor
t_{tr}	Transit time at which recombination between electron and holes ceases
$t_{\mathrm{tr}}^{\mathrm{eff}}$	Effective transit time at which recombination between electron and holes ceases in the RC-dominated limit
U	Toal potential difference across a diode
V_{app}	Voltage applied to a circuit containing a diode
V_{bi}	Built-in voltage
V_{drop}	Voltage drop across a diode in a circuit
V_{L}	Voltage measured across a load resistance
V_{oc}	Open-circuit voltage
x	Position in a one-dimensional device
$\bar{x}$	Position normalisation factor

Acronyms

CE	Charge extraction
HOMO	Highest occupied molecular orbital
LUMO	Lowest unoccupied molecular orbital
QFLS	Quasi-Fermi level splitting (also ΔE_{QFLS})
RC	Resistance-capacitance (effects)

S4. Energetic Landscape

To model the generation and transportation of charge carriers in a photovoltaic device, a drift-diffusion model was utilised in this work, wherein a semiconductor layer of energy gap E_{g} is sandwiched between two metallic electrodes called the anode (at position $x = 0$) and the cathode (at $x = d$, the active layer thickness). As illustrated in **Figure S2**, relative to the vacuum level E_{vac} these electrodes are described by the work-functions $\Phi_{\mathrm{an}} = E_{\mathrm{vac}} - E_{\mathrm{F,an}}$ and $\Phi_{\mathrm{cat}} = E_{\mathrm{vac}} - E_{\mathrm{F,cat}}$, respectively, where $E_{\mathrm{F,an}}$ is the Fermi level at the anode and $E_{\mathrm{F,cat}}$ is Fermi level at the cathode. The semiconducting material, on the other hand, ideally consists of two bands of continuous states; when no external effects are present (i.e., in thermodynamic equilibrium), all states in the lower band (the *valence band*) are occupied with electrons up to the valence level (E_{v}). While in the upper band (the *conduction band*), all states are occupied with 'holes' – i.e., a lack of electrons – down to the conduction level (E_{c}), which then defines the energy gap associated with the region devoid of occupied states as:[6]

$$E_{\mathrm{g}} = E_{\mathrm{c}} - E_{\mathrm{v}}. \quad \textbf{(S4.1)}$$

Alternatively, the energetic levels of the semiconductor may be defined relative to the vacuum level; the semiconductor has electron affinity χ_{aff} and ionisation potential χ_{IP}, where the former quantifies how much energy is required to remove an electron from the conduction band and free it to continuum ($\rightarrow E_{\mathrm{vac}}$) while the latter quantifies how much energy is needed to liberate an electron from the conduction band. Using these parameters, the bandgap can also be expressed as $E_{\mathrm{g}} = \chi_{\mathrm{IP}} - \chi_{\mathrm{aff}}$. In organic semiconductor blends, the transport of holes through the highest-occupied molecular orbitals (HOMOs) of the donor mimics the behaviour of the valence band in inorganic semiconductors, while the transport of electrons through the lowest-unoccupied molecular orbitals (LUMOs) of the acceptor is analogous to the conduction band in inorganic semiconductors.[11]

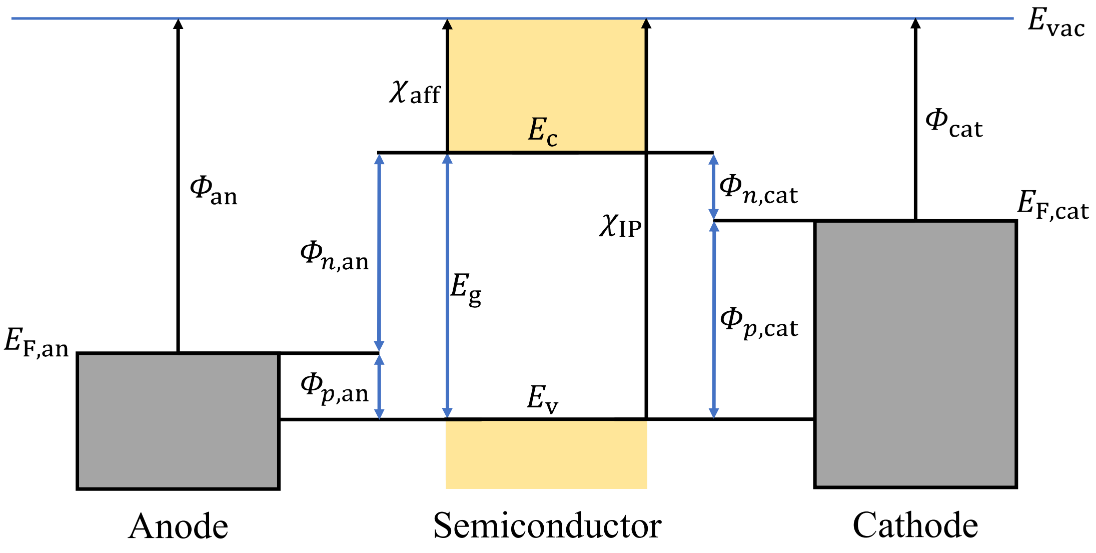

Figure S2: The energetic structure of a diode consisting of a semiconducting material of energetic gap E_g sandwiched between two metallic electrodes with work functions Φ_{an} and Φ_{cat}, respectively, resulting in electron and hole injection barriers at the anode ($\Phi_{n,an}$ and $\Phi_{p,an}$, respectively) and the cathode ($\Phi_{n,cat}$ and $\Phi_{p,cat}$, respectively).

At the anode-semiconductor contact, the injection barrier electrons must overcome to move from one material to the other is defined by $\Phi_{n,an} = \Phi_{an} - \chi_{aff}$, while the corresponding injection barrier for holes is defined by $\Phi_{p,an} = \chi_{IP} - \Phi_{an}$. Similarly, at the semiconductor-cathode contact, electrons are characterised by an injection barrier $\Phi_{n,cat} = \Phi_{cat} - \chi_{aff}$ while holes are described by an injection barrier $\Phi_{p,cat} = \chi_{IP} - \Phi_{cat}$. The materials used to form the anode and cathode are therefore chosen according to their work-functions Φ_{an} and Φ_{cat} to ensure ideal injection for a given application.

When the components of the diode are brought into contact, the excess of electrons in the cathode and holes in the anode give rise to concentration gradients that initially drive the diffusion of charge carriers into the semiconductor. This diffusion of electrons (while ions remain fixed) gives rise to an electric field across the semiconductor that results in the drift of charged electrons and holes in the opposite direction. If no external fields are applied to the device (in the dark), thermodynamic equilibrium is eventually reached

as a balance between counteracting drift and diffusion processes is established. The associated electric field can be thought of as inducing a tilt in the vacuum level, as illustrated in **Figure S3**, such that the Fermi level is constant across the device:

$$E_{\mathrm{F,an}} = E_{\mathrm{F,cat}} = E_{\mathrm{F}}, \quad \textbf{(S4.2)}$$

where E_{F} is the Fermi level of the semiconductor under thermodynamic equilibrium – i.e., when the device is in the dark and there is no applied voltage bias, resulting in no quasi-Fermi level splitting (QFLS). As a result, the energies of the conduction and valence bands, along with the vacuum level, vary as a function of position x in the semiconductor layer and are related to the electric potential $\psi(x,t)$ at time t via

$$\begin{aligned} E_{\mathrm{vac}}(x,t) - E_{\mathrm{vac}}(0,t) &= E_{\mathrm{c}}(x,t) - E_{\mathrm{c}}(0,t) = E_{\mathrm{v}}(x,t) - E_{\mathrm{v}}(0,t) \\ &= -q[\psi(x,t) - \psi(0,t)], \end{aligned} \quad \textbf{(S4.3)}$$

where $\psi(0,t)$ is some arbitrary reference level for the electric potential. The work-function, electron affinities, ionisation potentials, injection barriers, and the energy gap all remain independent of position. Consequently, a built-in voltage (V_{bi}) forms across the semiconductor of thickness d in the direction perpendicular to the contacts. This built-in voltage is defined by the difference in the vacuum level from the anode-semiconductor contact to the semiconductor-cathode contact, which can be rewritten in terms of the work functions of the electrodes as

$$qV_{\mathrm{bi}} = \Phi_{\mathrm{an}} - \Phi_{\mathrm{cat}}. \quad \textbf{(S4.4)}$$

Consequently, if two materials with similar work functions were used for the anode and cathode (or even the same material), the built-in voltage would effectively be nought.

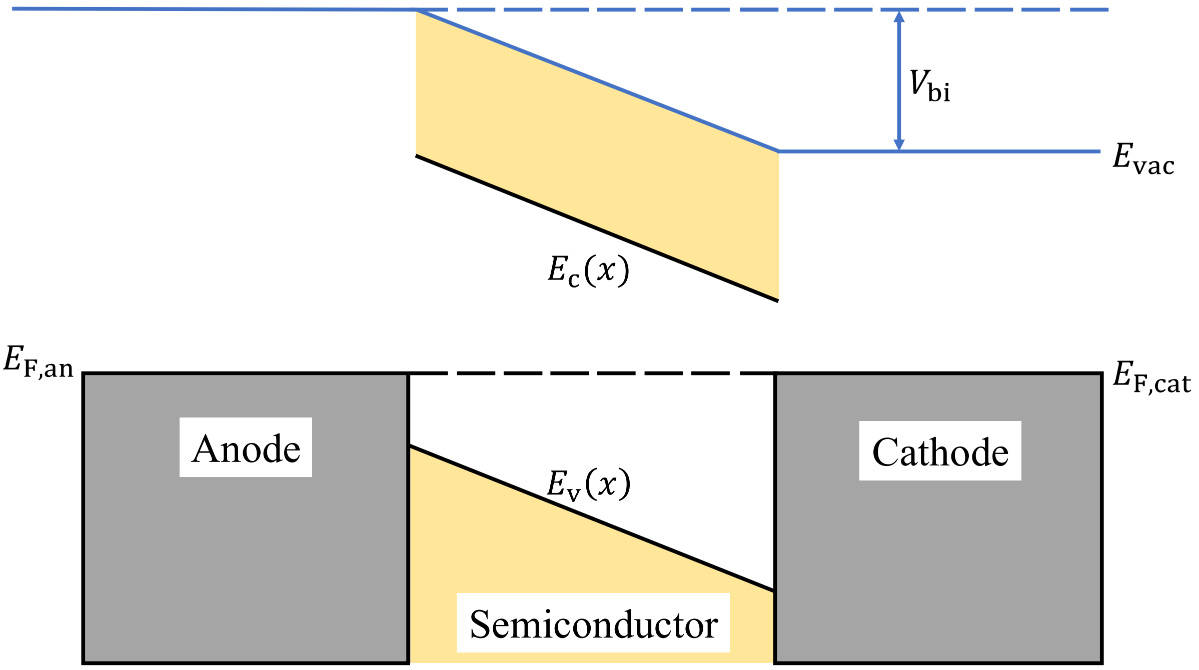

Figure S3: An illustration of the band-structure of the semiconductor-electrode sandwich once the constituent components have been brought into contact and a thermdoynamic equilibrium has been reached between counteracting drift and diffusion processes.

To disrupt the equilibrium and induce a current density $J(t)$ within the device, a voltage can be applied to it from an external source using the general circuit illustrated in **Figure S4**. In this circuit, a voltage $V_{app}(t)$ is applied using some variable voltage source. However, due to the finite series and load resistance of the external circuit, denoted by R_s and R_L respectively, the actual voltage drop across the device, $V_{drop}(t)$, will generally differ from the voltage applied to the circuit. The inclusion of the effect of the external circuit is herein referred to as the inclusion of 'resistance-capacitance (RC) effects'. For a device with (electrical) cross-sectional area A, the actual voltage drop across the diode relates to the voltage applied to the external circuit via

$$V_{drop}(t) = V_{app}(t) - J(t)A[R_s + R_l]. \quad \textbf{(S4.5)}$$

Hence, in the presence of some externally-applied voltage, the total potential difference across the diode, $U(t)$, is given by

$$U(t) = V_{\mathrm{drop}}(t) - V_{\mathrm{bi}}. \qquad \textbf{(S4.6)}$$

Note that in the case that either (i) the device is at open-circuit conditions (and $J(t) = 0$) and/or (ii) the series and load resistances are negligible, $V_{\mathrm{drop}}(t) = V_{\mathrm{app}}(t)$. Accounting for this potential difference, the current density induced by the transport of charge carriers in a photovoltaic device at any given applied voltage can be modelled using the equations and methods described in the next section.

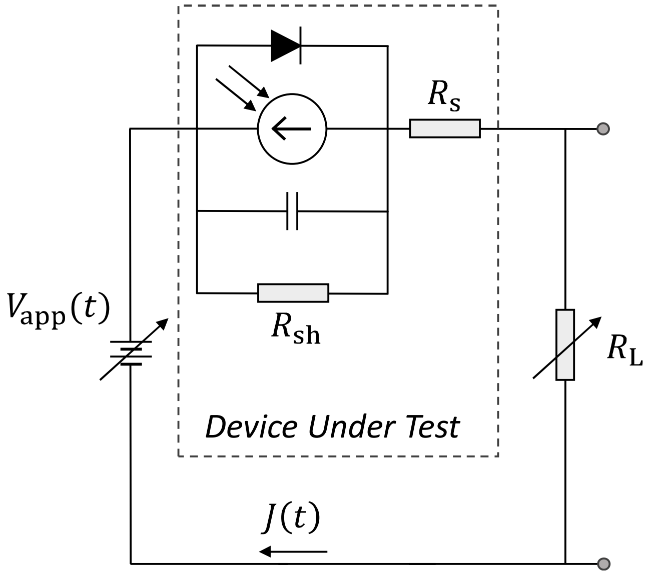

Figure S4: An equivalent circuit model describing a diode with (electrical) cross-sectional area A and shunt resistance R_{sh} in a circuit that experiences a voltage drop $V_{\mathrm{drop}}(t)$ producing a current density $J(t)$ at time t. The series and load resistance of the external circuit are denoted by R_{s} and R_{L} respectively, while the voltage applied to the external circuit, which doesn't necessarily equal the voltage drop across the device, is denoted by $V_{\mathrm{app}}(t)$. The current generated by the diode is determined through to the voltage measured across the load resistance, $V_{\mathrm{L}}(t)$, via Ohm's law.

S5. The System of Differential Equations

The drift-diffusion model utilized in this work involves iteratively solving a system of coupled differential equations in a finite element method. These equations are in congruence with Maxwell's equations; the series of coupled equations that describe electric ($\vec{\boldsymbol{F}}$) and magnetic ($\vec{\boldsymbol{B}}$) fields at position $\vec{\boldsymbol{r}}$ and time t in a medium with relative permittivity ϵ_r and relative permeability μ_r:[4, 12]

Gauss' Law:
$$\vec{\nabla}\cdot\vec{\boldsymbol{E}}(\vec{\boldsymbol{r}},t)=\frac{\rho(\vec{\boldsymbol{r}},t)}{\epsilon_\mathrm{r}\epsilon_0} \qquad \textbf{(S5.1)}$$

Gauss' Law for Magnetism:
$$\vec{\nabla}\cdot\vec{\boldsymbol{B}}(\vec{\boldsymbol{r}},t)=0 \qquad \textbf{(S5.2)}$$

Faraday's Law of Induction:
$$\vec{\nabla}\times\vec{\boldsymbol{E}}(\vec{\boldsymbol{r}},t)=-\frac{\partial\vec{\boldsymbol{B}}(\vec{\boldsymbol{r}},t)}{\partial t} \qquad \textbf{(S5.3)}$$

Ampère's Circuital Law:
$$\vec{\nabla}\times\vec{\boldsymbol{B}}(\vec{\boldsymbol{r}},t)=\mu_\mathrm{r}\mu_0\vec{\boldsymbol{J}}(\vec{\boldsymbol{r}},t) \qquad \textbf{(S5.4)}$$

The constants ϵ_0 and μ_0 are the permittivity and permeability of free space (vacuum), respectively, while $\vec{\boldsymbol{J}}(\vec{\boldsymbol{r}},t)$ is the total current density flowing through the device, which is defined by

$$\vec{\boldsymbol{J}}(\vec{\boldsymbol{r}},t)=\vec{\boldsymbol{J}}_\mathrm{C}(\vec{\boldsymbol{r}},t)+\epsilon_\mathrm{r}\epsilon_0\frac{\partial\vec{\boldsymbol{F}}(\vec{\boldsymbol{r}},t)}{\partial t}, \qquad \textbf{(S5.5)}$$

where $\vec{\boldsymbol{J}}_\mathrm{C}(\vec{\boldsymbol{r}},t)=\vec{\boldsymbol{J}}_n(\vec{\boldsymbol{r}},t)+\vec{\boldsymbol{J}}_p(\vec{\boldsymbol{r}},t)$ is the conduction current density composed of the sum of the current densities of electrons and holes, $\vec{\boldsymbol{J}}_n(\vec{\boldsymbol{r}},t)$ and $\vec{\boldsymbol{J}}_p(\vec{\boldsymbol{r}},t)$ respectively.

When considering materials with low dielectric constants, such as organic semiconductors,[11] magnetic phenomena can be considered as virtually negligible. In this case, the electric field may be expressed solely as the gradient of a (scalar) electric potential ψ:[13]

$$\vec{\boldsymbol{F}}(\vec{\boldsymbol{r}},t)=-\vec{\nabla}\psi(\vec{\boldsymbol{r}},t). \qquad \textbf{(S5.6)}$$

Combining this with *Gauss' Law* given in Equation **(S5.1)**, the first of the system of equations to be solved, *Poisson's equation*, can be used to relate the electric potential to the total space-charge density ρ via

$$\nabla^2\psi(\vec{\boldsymbol{r}},t) = -\frac{\rho(\vec{\boldsymbol{r}},t)}{\epsilon_\mathrm{r}\epsilon_0}, \qquad \textbf{(S5.7)}$$

where $\nabla^2 = \frac{\partial^2}{\partial x^2} + \frac{\partial^2}{\partial y^2} + \frac{\partial^2}{\partial z^2}$.

The next set of equations are *the continuity equations*, which describe the rate of change in the population of charge carriers (with respect to time) using the current densities flowing in to and out of the device, as well as the net generation and recombination rates across it. These equations are derived by first taking the divergence of Equation **(S5.4)**, *Ampère's Circuital Law*, then making use of the fact that the divergence of a curl vanishes,[12] such that

$$\vec{\nabla}\cdot\left(\vec{\boldsymbol{J}}_\mathrm{C}(\vec{\boldsymbol{r}},t) + \epsilon_r\epsilon_0\frac{\partial\vec{\boldsymbol{F}}(\vec{\boldsymbol{r}},t)}{\partial t}\right) = 0. \qquad \textbf{(S5.8)}$$

Making use of the commutativity of the derivative, as well as *Gauss' Law* given in Equation **(S5.1)**, one finds that the divergence of the conduction current density relates to the rate of change in space charge density via

$$\vec{\nabla}\cdot\vec{\boldsymbol{J}}_\mathrm{C}(\vec{\boldsymbol{r}},t) = -\frac{\partial\rho(\vec{\boldsymbol{r}},t)}{\partial t}. \qquad \textbf{(S5.9)}$$

For the work describe here, a one-dimensional drift-diffusion model is ample. We therefore assume the motion of electrons and holes to be the $\hat{x}$-direction, such that the divergence of the conduction current density reduces to $\vec{\nabla}\cdot\vec{\boldsymbol{J}}_\mathrm{C}(\vec{\boldsymbol{r}},t) = \frac{\partial J(x,t)}{\partial x}$. Consequently, in terms of the electron and hole current densities ($J_n(x,t)$ and $J_p(x,t)$, respectively), Equation **(S5.9)** may be rewritten as

$$\frac{\partial}{\partial x}\left[J_n(x,t) + J_p(x,t)\right] = -q\frac{\partial}{\partial t}[p(x,t) - n(x,t)], \qquad \textbf{(S5.10)}$$

where q is the elementary charge, and the space-charge density $\rho(x,t)$ at position x and time t is determined by the sum of total hole and electron densities ($p(x,t)$ and $n(x,t)$, respectively), $\rho(x,t) = q[p(x,t) - n(x,t)]$ (note that the relative sign difference between the two arises from the fact that electrons

have charge $-q$, and holes have charge $+q$). This equation may now be broken into two very similar equations. The first of these is *the continuity equation for electrons*:

$$\frac{\partial n(x,t)}{\partial t} = \frac{1}{q}\frac{\partial J_n(x,t)}{\partial x} + G(x,t) - \mathcal{R}(x,t). \qquad \textbf{(S5.11)}$$

While *the continuity equation for holes* reads as:

$$\frac{\partial p(x,t)}{\partial t} = -\frac{1}{q}\frac{\partial J_p(x,t)}{\partial x} + G(x,t) - \mathcal{R}(x,t) \qquad \textbf{(S5.12)}$$

In these equations, the terms $G(x,t)$ and $\mathcal{R}(x,t)$ denote the net charge generation and recombination rates, respectively. These rates are explored in closer detail shortly (in **Section S6**).

The electron and hole current densities are governed by the drift-diffusion equations. Therefore, the flow of both electrons and holes will constitute a sum of two contributions: (i) a drift motion that is driven by the electric field across the device, and (ii) a diffusive motion that is driven by gradients in the charge carrier densities. In general, the drift motion induces a drift current density $\vec{\boldsymbol{J}}_{\text{drift}}(\vec{\boldsymbol{r}},t)$ which is related to the electric field across the device $\vec{\boldsymbol{F}}(\vec{\boldsymbol{r}},t)$ via[14, 15]

$$\vec{\boldsymbol{J}}_{\text{drift}}(\vec{\boldsymbol{r}},t) = \sigma(\vec{\boldsymbol{r}},t)\vec{\boldsymbol{F}}(\vec{\boldsymbol{r}},t), \qquad \textbf{(S5.13)}$$

where $\sigma(\vec{\boldsymbol{r}},t)$ is the associated conductivity. While the diffusive motion gives rise to a diffusion current density $\vec{\boldsymbol{J}}_{\text{diff}}$ that relates to the charge density $\rho(\vec{\boldsymbol{r}},t)$ via Fick's Law[16, 17]

$$\vec{\boldsymbol{J}}_{\text{diff}}(\vec{\boldsymbol{r}},t) = D(\vec{\boldsymbol{r}},t)\vec{\boldsymbol{\nabla}}\rho(\vec{\boldsymbol{r}},t). \qquad \textbf{(S5.14)}$$

Here, $D(\vec{\boldsymbol{r}},t)$ is the diffusion coefficient at a given position $\vec{\boldsymbol{r}}$ and time t in the device – this, however, is usually taken as independent of both in an isotropic semiconducting material. For electrons, the total conduction current density is therefore,

$$J_n(x,t) = \mu_n(x,t)n(x,t)\frac{\partial E_{\text{F},n}(x,t)}{\partial x} = q\mu_n(x,t)n(x,t)F(x,t) + qD_n(x,t)\frac{\partial n(x,t)}{\partial x}, \qquad \textbf{(S5.15)}$$

where $E_{\mathrm{F},n}(x,t)$ is the quasi-Fermi level of electrons in the conduction band. The first term on the right-hand side is the electron drift current, where $\mu_n(x,t)$ is the electron mobility and F, the electric field across the device, is defined by reducing Equation **(S5.6)** to one spatial dimension:

$$F(x,t) = -\frac{\partial\psi(x,t)}{\partial x}. \tag{S5.16}$$

The right-most term in Equation **(S5.15)** is the electron diffusion current, with $D_n(x,t)$ being the associated diffusion coefficient for electrons. We herein assume that the Boltzmann approximation applies, such that $D_n(x,t)$ can be related to the electron mobility via the classical Einstein relation:

$$D_n(x,t) = \mu_n(x,t)\frac{k_{\mathrm{B}}T}{q}, \tag{S5.17}$$

where k_{B} is the Boltzmann constant and T is temperature. Through the same reasoning, the hole current density is given by

$$J_p(x,t) = \mu_p(x,t)p(x,t)\frac{\partial E_{\mathrm{F},p}(x,t)}{\partial x} = q\mu_p(x,t)p(x,t)F(x,t) - qD_p(x,t)\frac{\partial p(x,t)}{\partial x}, \tag{S5.18}$$

where $E_{\mathrm{F},p}$ is the quasi-Fermi level for the holes in the valence band, while $\mu_p(x,t)$ and $D_p(x,t)$ are the hole mobility and diffusion coefficient, respectively; as with the electrons, the two are assumed to be related via the classical Einstein relation. We reiterate that carrier mobilities (and diffusion coefficients) are typically considered to be independent of position and time.

Note that upon solving Equation **(S5.15)** for $n(x,t)$, while making use of Equations **(S5.16)** and **(S5.17)**, the electron density can be expressed as

$$n(x,t) = n_{\mathrm{c}}\exp\left(\frac{E_{\mathrm{F},n}(x,t) - E_{\mathrm{c}}(x,t)}{k_{\mathrm{B}}T}\right). \tag{S5.19}$$

where n_{c} is a constant pre-factor corresponding to the *effective density of free electron states*. Similarly, solving Equation **(S5.18)** for $p(x,t)$, the hole density is found as

$$p(x,t) = n_\mathrm{v} \exp\left(\frac{E_\mathrm{v}(x,t) - E_{\mathrm{F},p}(x,t)}{k_\mathrm{B}T}\right), \tag{S5.20}$$

where n_v is the corresponding *effective density of free hole states.* Taking the product of the electron and hole densities (where the QFLS is given by $\Delta E_\mathrm{QFLS} = E_{\mathrm{F},n} - E_{\mathrm{F},p}$), one obtains

$$np = n_\mathrm{c} n_\mathrm{v} \exp\left(\frac{E_{\mathrm{F},n} - E_\mathrm{c} + E_\mathrm{v} - E_{\mathrm{F},p}}{k_\mathrm{B}T}\right) = n_\mathrm{int}^2 \exp\left(\frac{\Delta E_\mathrm{QFLS}}{k_\mathrm{B}T}\right), \tag{S5.21}$$

where the parameter $n_\mathrm{int} \equiv \sqrt{n_\mathrm{c} n_\mathrm{v}} \exp\left(-\frac{E_\mathrm{g}}{2k_\mathrm{B}T}\right)$ is referred to as the intrinsic charge carrier density, while product of the carrier densities depends exponentially on the QFLS. At thermodynamic equilibrium, the intrinsic carrier density therefore relates to the equilibrium carrier densities (denoted as $n_\mathrm{eq}(x)$ and $p_\mathrm{eq}(x)$) via

$$n_\mathrm{int}^2 = n_\mathrm{eq}(x) p_\mathrm{eq}(x), \tag{S5.22}$$

which is known as the *law of mass action*.

S6. Generation and Recombination

In general, the generation rate G of free charge carriers in the bulk is composed of both thermal generation and external (optical) photogeneration. The thermal generation is always present within the semiconductor material, even in thermal equilibrium. The recombination rate $\mathcal{R}(x,t)$ at a particular position and time within the bulk, on the other hand, depends on the local electron and hole densities. For bimolecular recombination, we have

$$\mathcal{R}(x,t) = \beta(x,t)n(x,t)p(x,t). \tag{S6.1}$$

Herein, the bimolecular recombination rate constant β may be expressed in terms of intrinsic material parameters as[18, 19]

$$\beta(x,t) = \gamma\beta_{\mathrm{L}}(x,t) = \gamma\frac{q\left[\mu_n(x,t)+\mu_p(x,t)\right]}{\epsilon_{\mathrm{r}}\epsilon_0}, \tag{S6.2}$$

where $\beta_{\mathrm{L}} = \frac{q(\mu_n+\mu_p)}{\epsilon_{\mathrm{r}}\epsilon_0}$ is the Langevin encounter rate constant, which is the rate at which free electrons and holes encounter each other in the semiconductor. Not every encounter between the two species results in a recombination event, however, and so the recombination rate constant is reduced by the Langevin reduction factor γ, which is typically on the order of 10^{-1} to 10^{-3} for organic materials.[20, 21] Furthermore, the thermal generation rate can be obtained by balancing generation and recombination at thermodynamic equilibrium such that $G = \beta n_{\mathrm{eq}}(x)p_{\mathrm{eq}}(x) = \beta n_{\mathrm{int}}^2$, in accordance with Equation **(S5.22)**. In the general case, the net generation-recombination rate of free electrons and holes will be

$$G(x,t) - \mathcal{R}(x,t) = G_{\mathrm{light}}(x,t) - \beta(x,t)\left[n(x,t)p(x,t) - n_{\mathrm{int}}^2\right]. \tag{S6.3}$$

Here, $G_{\mathrm{light}}(x,t)$ denotes the additional photogeneration rate induced when the device is illuminated by an external light source. The exact nature of $G_{\mathrm{light}}(x,t)$ is usually taken as spectrally-flat, exponentially-decaying in a Beer-Lambert-like profile, or it can have a far more complex profile and lineshape if optical interference effects are accounted for, e.g., via the use of an optical transfer-matrix model under a given

light source.[22-24] In this case, the exact profile of the photo-generation rate will depend on the circumstances being considered, including the used device architecture and the angle of incidence of the light.

S7. Boundary Conditions

To numerically solve the set of coupled differential equations presented in **Section S5** (using the generation and recombination rates specified in **Section S6**), the boundary conditions that limit the electric potential and carrier densities must be known. First, we consider the boundary conditions on the electric potential, which are determined by the total potential difference $U(t)$ across the diode (calculated using Equation **(S4.4)** to **(S4.6)**), in conjunction with the fact that the electric field across the device satisfies $U(t) = \int_0^d F(x,t)\mathrm{d}x$. It follows from Equation **(S5.16)** that the electric potential satisfies the following boundary condition,

$$\psi(0,t) - \psi(d,t) = U(t) = V_{\mathrm{drop}}(t) - V_{\mathrm{bi}}. \qquad \textbf{(S7.1)}$$

As gradients in electric potential drive motion, not the magnitude of the potential itself, the potential at $x = 0$ is usually set to nought, $\psi(0,t) = 0$, such that the electric potential at $x = d$ is given by $\psi(d,t) = V_{\mathrm{bi}} - V_{\mathrm{drop}}(t)$. Using the relation between the energy of the vacuum level E_{vac} and the electric potential given in Equation **(S4.3)**, the total potential difference across the diode is equivalent to

$$qU(t) = E_{\mathrm{vac}}(d,t) - E_{\mathrm{vac}}(0,t) = E_{\mathrm{c}}(d,t) - E_{\mathrm{c}}(0,t) = E_{\mathrm{v}}(d,t) - E_{\mathrm{v}}(0,t). \qquad \textbf{(S7.2)}$$

Making use of the definition of the built-in voltage, the splitting in the Fermi-levels of the anode and cathode can be related to the voltage drop across the diode at a particular time:

$$qV_{\mathrm{drop}}(t) = (E_{\mathrm{vac}}(d,t) - \Phi_{\mathrm{cat}}) - (E_{\mathrm{vac}}(0,t) - \Phi_{\mathrm{an}}) = E_{\mathrm{F,cat}} - E_{\mathrm{F,an}}. \qquad \textbf{(S7.3)}$$

Note that in thermal equilibrium, there is no voltage drop across device and the electron and hole currents are both identically zero; hence $E_{\mathrm{F,cat}} = E_{\mathrm{F,an}} = E_{\mathrm{F},n} = E_{\mathrm{F},p}$ in this case, as expected.

Next, we look to the boundary conditions that govern the electron and hole carrier densities. With the electron and hole densities written in terms of their respective Fermi levels in Equation **(S5.19)** and **(S5.20)**, the carrier densities at the contacts can be determined by assuming that – just at the contacts – the

quasi-Fermi levels are in equilibrium with the Fermi levels of the electrodes. Making use of the relative energy levels gives the boundary conditions on the carrier densities:

$$n(x=0) = n_{\mathrm{an}} = n_{\mathrm{c}} \exp\left(-\frac{\Phi_{n,\mathrm{an}}}{k_{\mathrm{B}}T}\right), \quad \textbf{(S7.4a)}$$

$$n(x=d) = n_{\mathrm{cat}} = n_{\mathrm{c}} \exp\left(-\frac{\Phi_{n,\mathrm{cat}}}{k_{\mathrm{B}}T}\right), \quad \textbf{(S7.4b)}$$

$$p(x=0) = p_{\mathrm{an}} = n_{\mathrm{v}} \exp\left(-\frac{\Phi_{p,\mathrm{an}}}{k_{\mathrm{B}}T}\right), \quad \textbf{(S7.4c)}$$

$$p(x=d) = p_{\mathrm{cat}} = n_{\mathrm{v}} \exp\left(-\frac{\Phi_{p,\mathrm{cat}}}{k_{\mathrm{B}}T}\right). \quad \textbf{(S7.4d)}$$

Here, n_{an} and p_{an} are the electron and hole densities at the anode, respectively, while n_{cat} and p_{cat} are the corresponding densities at the cathode, respectively.

S8. Normalisation

To reduce the computational demand of the drift-diffusion simulations, the parameters are normalised to more manageable values in the following discussion. A summary of the normalised parameters and their normalisation factors is provided in **Table S4.** To start, we normalise the electric potential with the normalisation factor $\bar{\psi} = \frac{k_B T}{q}$ and we normalise the position in the active layer to its thickness, d, such that Poisson's equation may be rewritten as

$$\frac{\partial^2 \psi'}{\partial x'^2} = n' - p', \quad \textbf{(S8.1)}$$

where the electron and hole densities have both been with normalisation factor $\bar{n} = \frac{\epsilon_r \epsilon_0 \bar{\psi}}{q d^2}$. Note that the normalised parameters are labelled with primes throughout, while the normalisation factors are denoted with overhead bars. In turn, the current density equations discussed in Equation **(S5.15)-(S5.18)** can be written in a normalised form as

$$J_n' = \mu_n' \left(-n' \frac{\partial \psi'}{\partial x'} + \frac{\partial n'}{\partial x'} \right), \quad \textbf{(S8.2a)}$$

$$J_p' = -\mu_p' \left(p' \frac{\partial \psi'}{\partial x'} + \frac{\partial p'}{\partial x'} \right). \quad \textbf{(S8.2b)}$$

Here, the electron and hole mobilities have been normalised to $\bar{\mu} = 10^{-3} \text{cm}^2 \text{V}^{-1} \text{s}^{-1}$, while the current densities have been normalised to $\bar{J} = \frac{\bar{n} \bar{\mu} k_B T}{d}$. Looking now to the continuity equations given in Equation **(S5.11)** and **(S5.12)**, normalising the generation and recombination rates to $\bar{G} = \frac{\bar{J}}{q d}$ and the time to $\bar{t} = \frac{\bar{n}}{\bar{G}}$ yields

$$\frac{\partial n'}{\partial t'} = \frac{\partial J_n'}{\partial x'} + G' - \mathcal{R}', \quad \textbf{(S8.3a)}$$

$$\frac{\partial p'}{\partial t'} = -\frac{\partial J_p'}{\partial x'} + G' - \mathcal{R}'. \quad \textbf{(S8.3b)}$$

With the key formulae now normalised, the expressions may be discretised with respect to time and space, enabling the numerical evaluation of the carrier densities, the electric potential, and all other parameters.

Table S4: A summary of the key parameters used to numerically solve the drift-diffusion equations, alongside their normalised forms and their normalisation factors.

Parameter	Normalised Form	Normalisation Factor
x	$x' = \frac{x'}{\bar{x}}$	$\bar{x} = d$
ψ	$\psi' = \frac{\psi}{\bar{\psi}}$	$\bar{\psi} = \frac{k_B T}{q}$
n	$n' = \frac{n}{\bar{n}}$	$\bar{n} = \frac{\epsilon_r \epsilon_0 \bar{\psi}}{q d^2}$
p	$p' = \frac{p}{\bar{n}}$	
μ_n	$\mu_n' = \frac{\mu_n}{\bar{\mu}}$	$\bar{\mu} = 10^{-3} \mathrm{cm^2 V^{-1} s^{-1}}$
μ_p	$\mu_p' = \frac{\mu_p}{\bar{\mu}}$	
J_n	$J_n' = \frac{J_n}{\bar{J}}$	$\bar{J} = \frac{k_B T \bar{n} \bar{\mu}}{d}$
J_p	$J_p' = \frac{J_p}{\bar{J}}.$	
G	$G' = \frac{G}{\bar{G}}$	$\bar{G} = \frac{\bar{J}}{qd}$
$\mathcal{R}$	$\mathcal{R}' = \frac{\mathcal{R}}{\bar{G}}$	
t	$t' = \frac{t}{\bar{t}}$	$\bar{t} = \frac{\bar{n}}{\bar{G}}$

S9. Discretising the Drift-Diffusion Equations

With the parameters normalised using the prescription outlined in the previous section, the equations describing the drift-diffusion model summarised in **Section S5** are now discretised with respect to time and position in the device. As illustrated in **Figure S5**, the semiconductor layer of thickness d may be discretised into $N+1$ slivers of width $\delta = \frac{d}{N+1}$, or, in normalised units,

$$\delta' = \frac{\delta}{\bar{x}} = \frac{1}{N+1}. \quad \textbf{(S9.1)}$$

Discretising the semiconductor layer in this way enables the numerical evaluation of the normalised electric potential, ψ', and the normalised carrier densities, n' and p', at the $N+2$ points that lie between the slivers (including at the contacts, where $x = 0$ and $x = d$). Each of these points is attributed an index $j \in \{0,1,\ldots,N+1\}$, such that a particular normalised position in the semiconductor relates to its index via

$$x'_j = j\delta'. \quad \textbf{(S9.2)}$$

To simplify notation, the electric potential at this point is denoted as $\psi'_{j,k} = \psi'(x'_j, t'_k)$, whilst the electron density and hole densities are labelled as $n'_{j,k} = n'(x'_j, t'_k)$ and $p'_{j,k} = p'(x'_j, t'_k)$. Here, k denotes the index of the time in a *transient* simulation. As the electric field and current densities depend on the first derivatives of ψ', n', and p', for enhanced accuracy these will be evaluated in discretised form halfway between points with index j; these quantities are therefore labelled with position index $j + \frac{1}{2}$.

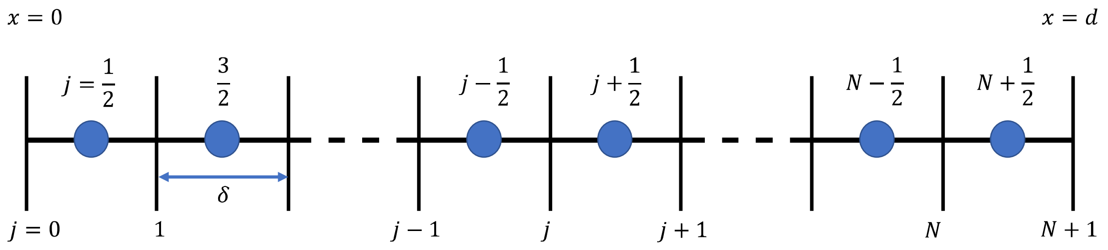

Figure S5: Discretisation of the active semiconductor layer of thickness d into $N+1$ slivers of width δ, or normalised width $\delta' = \frac{\delta}{d}$. The resultant $N+2$ points between before and after the slivers (including the

contacts at $x = 0$ and $x = d$) are labelled with index j. Positions in the centre of the slivers are labelled with index $j + \frac{1}{2}$.

To fully discretise the differential equations in the presented drift-diffusion model, the first and second derivatives of some parameters with respect to position will need to be discretised. For now, the time-dependence of these parameters is neglected. For Poisson's equation and the two continuity equations, the discretisation of these derivatives is based on the Taylor expansion, which is generally defined on a function $f(x)$ about a point a as[25]

$$f(x) = \sum_{n=0}^{\infty} \frac{(x-a)^n}{n!} \frac{\partial^n f(x)}{\partial x^n}\bigg|_{x=a} . \quad \textbf{(S9.3)}$$

The discretisation of the derivatives is now exemplified for the normalised electric potential, ψ', with the motive of discretising Poisson's equation. To determine the electric potential at a point with position index $j + \frac{1}{2}$, it is necessary to expand the electric potential about $x' = x'_{j+\frac{1}{2}} = x'_j + \frac{\delta'}{2}$, giving

$$\psi'_{j,k} = \psi'(x'_j, t'_k) = \sum_{n=0}^{\infty} \frac{\left(x'_j - x'_{j+\frac{1}{2}}\right)^n}{n!} \frac{\partial^n \psi'}{\partial x^n}\bigg|_{x=x'_{j+\frac{1}{2}}} . \quad \textbf{(S9.4)}$$

Substituting in for $x'_{j+\frac{1}{2}}$ yields

$$\psi'_{j,k} = \sum_{n=0}^{\infty} \frac{\left(-\frac{\delta'}{2}\right)^n}{n!} \frac{\partial^n \psi'}{\partial x^n}\bigg|_{x=x'_{j+\frac{1}{2}},t'_k} . \quad \textbf{(S9.5)}$$

Expanding out the first few terms and using indexed notation gives

$$\psi'_{j,k} = \psi'_{j+\frac{1}{2},k} - \left(\frac{\delta'}{2}\right)\frac{\partial \psi'}{\partial x'}\bigg|_{j+\frac{1}{2},k} + \left(\frac{\delta'^2}{8}\right)\frac{\partial^2 \psi'}{\partial x'^2}\bigg|_{j+\frac{1}{2},k} - \left(\frac{\delta'^3}{48}\right)\frac{\partial^3 \psi'}{\partial x'^3}\bigg|_{j+\frac{1}{2},k} + \cdots \quad \textbf{(S9.6)}$$

A similar expansion about $x' = x'_{j-\frac{1}{2}} = x'_j - \frac{\delta'}{2}$ yields

$$\psi'_{j,k} = \sum_{n=0}^{\infty} \frac{\left(\frac{\delta'}{2}\right)^n}{n!} \frac{\partial^n \psi'}{\partial x^n}\bigg|_{x=x'_{j-\frac{1}{2}},t'_k}$$
$$= \psi'_{j-\frac{1}{2},k} + \left(\frac{\delta'}{2}\right)\frac{\partial \psi'}{\partial x'}\bigg|_{j-\frac{1}{2},k} + \left(\frac{\delta'^2}{8}\right)\frac{\partial^2 \psi'}{\partial x'^2}\bigg|_{j-\frac{1}{2},k} + \left(\frac{\delta'^3}{48}\right)\frac{\partial^3 \psi'}{\partial x'^3}\bigg|_{j-\frac{1}{2},k} + \cdots \quad \textbf{(S9.7)}$$

Shifting the position index in Equation **(S9.7)** upwards by one gives the Taylor expansion for the electric potential at position index $j + 1$,

$$\psi'_{j+1,k} = \psi'_{j+\frac{1}{2},k} + \left(\frac{\delta'}{2}\right)\frac{\partial \psi'}{\partial x'}\bigg|_{j+\frac{1}{2},k} + \left(\frac{\delta'^2}{8}\right)\frac{\partial^2 \psi'}{\partial x'^2}\bigg|_{j+\frac{1}{2},k} + \left(\frac{\delta'^3}{48}\right)\frac{\partial^3 \psi'}{\partial x'^3}\bigg|_{j+\frac{1}{2},k} + \cdots \quad \textbf{(S9.8)}$$

Subtracting Equation **(S9.6)** from this and rearranging yields an expression for the discretised first derivative of the electric potential at position $j + \frac{1}{2}$:

$$\frac{\partial \psi'}{\partial x'}\bigg|_{j+\frac{1}{2},k} = \frac{\psi'_{j+1,k} - \psi'_{j,k}}{\delta'} - \mathcal{O}(\delta'^2) \approx \frac{\psi'_{j+1,k} - \psi'_{j,k}}{\delta'}. \quad \textbf{(S9.9)}$$

This, of course, is just a first order approximation for the derivative as the difference in the independent variable over the difference in the independent variable, noting that the terms on the order of $\delta'^2 \to 0$ for sufficiently-large N.

An expression for the second derivative of the electric potential at a position with index j (which is needed to discretise Poisson's equation) may be obtained by shifting Equation **(S9.9)** downwards by one position index:

$$\frac{\partial \psi'}{\partial x'}\bigg|_{j-\frac{1}{2},k} = \frac{\psi'_{j,k} - \psi'_{j-1,k}}{\delta'} - \mathcal{O}(\delta'^2) \approx \frac{\psi'_{j,k} - \psi'_{j-1,k}}{\delta'}. \quad \textbf{(S9.10)}$$

Subtracting this from Equation **(S9.9)** and rearranging gives an expression for discretised form of the second derivative of the electric potential:

$$\left.\frac{\partial^2\psi'}{\partial x'^2}\right|_{j,k} = \frac{\left.\frac{\partial\psi'}{\partial x'}\right|_{j+\frac{1}{2},k} - \left.\frac{\partial\psi'}{\partial x'}\right|_{j-\frac{1}{2},k}}{\delta'} - \mathcal{O}\left(\delta'^2\right) \approx \frac{\psi'_{j+1,k} - 2\psi'_{j,k} + \psi'_{j-1,k}}{\delta'^2}. \quad \textbf{(S9.11)}$$

Using this, the discretised Poisson equation can be written in its normalised form as

$$\frac{\psi'_{j+1,k} - 2\psi'_{j,k} + \psi'_{j-1,k}}{\delta'^2} = n'_{j,k} - p'_{j,k} \quad \textbf{(S9.12)}$$

Using this expression, the electric potential at a position with index j can be related to the carrier densities at that position, and the electric potential at the adjacent sites.

A similar discretisation can be applied to the continuity equations. Before this, the drift-diffusion relations need to be discretised separately. However, as the carrier densities generally vary strongly with position, a similar Taylor expansion is not feasible for the current density relations. Instead, we utilise the approach described by Scharfetter and Gummel.[26] We start by discretising the x-derivative of the current densities, where we neglect (for now) the time index k. To relate the electron current density to the discretised electron densities at points j and $j+1$, we solve Equation (**S8.2a**) within the interval $x'_j \leq x \leq x'_{j+1}$, by first multiplying it by the integrating factor $e^{-\psi'(x')}$ and rearranging to get

$$\frac{J'_n(x')}{\mu'_n(x')} e^{-\psi'(x')} = \left(-n'(x')\frac{\partial\psi'}{\partial x'} + \frac{\partial n'}{\partial x'}\right) e^{-\psi'(x')} = \frac{\partial\left[n'(x') e^{-\psi'(x')}\right]}{\partial x'}. \quad \textbf{(S9.13)}$$

Integrating this with respect to x' from x'_j to x'_{j+1} yields

$$\int_{x'_j}^{x'_{j+1}} \frac{J'_n(x')}{\mu'_n(x')} e^{-\psi'(x')}\,\mathrm{d}x' = n'\left(x'_{j+1}\right)e^{-\psi'\left(x'_{j+1}\right)} - n'\left(x'_j\right)e^{-\psi'\left(x'_j\right)}. \quad \textbf{(S9.14)}$$

By assuming that the current density, mobility, and first derivative of the potential vary minimally across this domain, such that $J'_n(x') \approx J'_n\big|_{j+\frac{1}{2}}$, $\mu'_n(x') \approx \mu'_n\big|_{j+\frac{1}{2}}$, and $\frac{\partial\psi'}{\partial x'} \approx \left.\frac{\partial\psi'}{\partial x'}\right|_{j+\frac{1}{2}}$, the integral can be evaluated, allowing for Equation **(S9.14)** to be further simplified as

$$\frac{J'_n\big|_{j+\frac{1}{2}}\delta'}{\mu'_n\big|_{j+\frac{1}{2}}}\left(\frac{e^{-\psi'_j}-e^{-\psi'_{j+1}}}{\psi'_{j+1}-\psi'_j}\right)=n'_{j+1}e^{-\psi'_{j+1}}-n'_je^{-\psi'_j}, \tag{S9.15}$$

where the first derivative of the electric potential was substituted in from Equation **(S9.9)**. Finally, rearranging Equation **(S9.15)** yields

$$J'_n\big|_{j+\frac{1}{2}}=\frac{\mu'_n\big|_{j+\frac{1}{2}}}{\delta'}\frac{\psi'_{j+1}-\psi'_j}{1-e^{\psi'_j-\psi'_{j+1}}}\left[n'_{j+1}e^{\psi'_j-\psi'_{j+1}}-n'_j\right]. \tag{S9.16}$$

Utilising the Bernoulli function, defined as $\mathcal{B}(y)=y/(e^y-1)$ with $e^y\mathcal{B}(y)=\frac{ye^y}{e^y-1}=\frac{-y}{e^{-y}-1}=\mathcal{B}(-y)$,[4] this expression can be rewritten as

$$J'_n\big|_{j+\frac{1}{2}}=\frac{\mu'_n\big|_{j+\frac{1}{2}}}{\delta'}\left[n'_{j+1}\mathcal{B}\left(\psi'_{j+1}-\psi'_j\right)-n'_j\mathcal{B}\left(\psi'_j-\psi'_{j+1}\right)\right]. \tag{S9.17}$$

Through an equally-detailed derivation, it can be shown that the hole current density at the point $x=x_{j+\frac{1}{2}}$ is given in discretised form by

$$J'_p\big|_{j+\frac{1}{2}}=\frac{\mu'_p\big|_{j+\frac{1}{2}}}{\delta'}\left[p'_j\mathcal{B}\left(\psi'_{j+1}-\psi'_j\right)-p'_{j+1}\mathcal{B}\left(\psi'_j-\psi'_{j+1}\right)\right]. \tag{S9.18}$$

In practice, we found that numerical evaluation of the Bernoulli function $\mathcal{B}(y)$ in the case of a small argument y is computationally expensive. To compute the Bernoulli function for $|y|<10^{-6}$, we employ a series approximation $\mathcal{B}(y)=\frac{y}{e^y-1}\approx 1-\frac{y}{2}+\frac{y^2}{12}-\frac{y^4}{720}$.

With the current densities written in their discretised forms in Equation **(S9.17)** and **(S9.18)**, the continuity equations can now be discretised. Firstly, the derivative of the electron current density at the point with position index j is determined using

$$\left.\frac{\partial J'_n}{\partial x'}\right|_j\approx\frac{J'_n\big|_{j+\frac{1}{2}}-J'_n\big|_{j-\frac{1}{2}}}{\delta'}. \tag{S9.19}$$

Substituting in the discretised form for the current density gives

$$\left.\frac{\partial J'_n}{\partial x'}\right|_j = a_{n,j}n'_{j-1} + b_{n,j}n'_j + c_{n,j}n'_{j+1}, \quad \textbf{(S9.20)}$$

where the coefficients on each of the terms are given by

$$a_{n,j} = \frac{\mu'_n|_{j-\frac{1}{2}}}{\delta'^2}\mathcal{B}(\psi'_{j-1} - \psi'_j), \quad \textbf{(S9.21a)}$$

$$b_{n,j} = -\frac{\mu'_n|_{j+\frac{1}{2}}}{\delta'^2}\mathcal{B}(\psi'_j - \psi'_{j+1}) - \frac{\mu'_n|_{j-\frac{1}{2}}}{\delta'^2}\mathcal{B}(\psi'_j - \psi'_{j-1}), \quad \textbf{(S9.21b)}$$

$$c_{n,j} = \frac{\mu'_n|_{j+\frac{1}{2}}}{\delta'^2}\mathcal{B}(\psi'_{j+1} - \psi'_j). \quad \textbf{(S9.21c)}$$

Similarly, the discretised form of the first derivative of the hole current density may be written as

$$\left.\frac{\partial J'_p}{\partial x'}\right|_j \approx \frac{J'_p|_{j+\frac{1}{2}} - J'_p|_{j-\frac{1}{2}}}{\delta'} = a_{p,j}p'_{j-1} + b_{p,j}p'_j + c_{p,j}p'_{j+1}, \quad \textbf{(S9.22)}$$

where the pre-factors are

$$a_{p,j} = -\frac{\mu'_p|_{j-\frac{1}{2}}}{\delta'^2}\mathcal{B}(\psi'_j - \psi'_{j-1}) \quad \textbf{(S9.23a)}$$

$$b_{p,j} = \frac{\mu'_p|_{j+\frac{1}{2}}}{\delta'^2}\mathcal{B}(\psi'_{j+1} - \psi'_j) + \frac{\mu'_p|_{j-\frac{1}{2}}}{\delta'^2}\mathcal{B}(\psi'_{j-1} - \psi'_j), \quad \textbf{(S9.23b)}$$

$$c_{p,j} = -\frac{\mu'_p|_{j+\frac{1}{2}}}{\delta'^2}\mathcal{B}(\psi'_j - \psi'_{j+1}). \quad \textbf{(S9.23c)}$$

With the x-derivative of the current densities in hand, we can now fully discretise the continuity equations by re-introducing the time index and approximating the t-derivative of the electron density at position index j and time index k with

$$\left.\frac{\partial n'}{\partial t'}\right|_{j,k} = \frac{n'_{j,k} - n'_{j,k-1}}{\tau'_k} - \mathcal{O}({\tau'_k}^2) \approx \frac{n'_{j,k} - n'_{j,k-1}}{\tau'_k}. \quad \textbf{(S9.24)}$$

Here $n'_{j,k-1}$ is the electron density at position j at the previous time index $k-1$, while $\tau'_k = \frac{\tau_k}{\bar{t}}$ is normalised time difference between the current time and the previous time: $\tau_k = t_k - t_{k-1}$. This timestep need not be fixed (simulating a 1 ms transient experiment at 1 ps timesteps is impractical, so the timestep could be increased as the simulation progresses), it must, however, be sufficiently small for the approximation on the right-hand side of Equation **(S9.24)** to be made. In a similar vein, the time-derivative of the hole density at a position with index j is given by

$$\left.\frac{\partial p'}{\partial t'}\right|_{j,k} \approx \frac{p'_{j,k} - p'_{j,k-1}}{\tau'_k}. \qquad \textbf{(S9.25)}$$

Such that the discretised continuity equations may now be rewritten as

$$\frac{n'_{j,k} - n'_{j,k-1}}{\tau'_k} \approx a_{n,j,k}n'_{j-1,k} + b_{n,j,k}n'_{j,k} + c_{n,j,k}n'_{j+1,k} + G'_{j,k} - \mathcal{R}'_{j,k}, \qquad \textbf{(S9.26a)}$$

$$\frac{p'_{j,k} - p'_{j,k-1}}{\tau'_k} \approx -a_{p,j,k}p'_{j-1,k} - b_{p,j,k}p'_{j,k} - c_{p,j,k}p'_{j+1,k} + G'_{j,k} - \mathcal{R}'_{j,k}. \qquad \textbf{(S9.26b)}$$

In the special case of a static simulation, the timestep $\tau'_k \to \infty$ and the left-hand sides of both these expressions tend to nought.

S10. Discretising the Voltage Drop (Including Resistance-Capacitance Effects)

With the continuity equations discretised, nearly all of the ingredients are prepared for numerically solving the equations of the drift diffusion model using the iterative approach described in the **Section S11**. For the general case of a transient simulation, however, it is necessary to first discretise the voltage drop across the device with respect to time because, as shown in Equation **(S4.5)**, $V_{\text{drop}}(t)$ depends on the current density produced by the device (in the case that RC effects are not negligible). To discretise the voltage drop, we first separate the total current density into two contributions:

$$J(t) = J_{\text{C}}(t) + J_{\text{D}}(t). \qquad \textbf{(S10.1)}$$

Here, $J_{\text{C}}(t)$ is the spatially-averaged conduction current density given by the sum of the spatially-averaged electron and hole current densities generated by the diode:[1]

$$J_{\text{C}}(t) = \frac{1}{d}\int_0^d \left[J_n(x,t) + J_p(x,t)\right] \text{d}x \approx \frac{\bar{J}}{N+1}\sum_{j=0}^{N}\left(J_n'\big|_{j+\frac{1}{2},k} + J_p'\big|_{j+\frac{1}{2},k}\right). \qquad \textbf{(S10.2)}$$

The right-hand side of this expression is the discretised form of the integral, and it has been written using the normalised current densities J_n' and J_p' (which have been re-scaled using $\bar{J}$). The other contribution to the total current density, $J_{\text{D}}(t)$, is the spatially-averaged displacement current density induced by changes in the voltage drop across the diode with respect to time. In a material with negligible polarisation, the displacement current may be given by[27]

$$J_{\text{D}}(t) = \frac{1}{d}\int_0^d \epsilon_{\text{r}}\epsilon_0 \frac{\partial F(x,t)}{\partial t} \text{d}x = C_0 \frac{\partial V_{\text{drop}}(t)}{\partial t} \approx \frac{C_0}{\tau_k}\left[V_{\text{drop}}\big|_k - V_{\text{drop}}\big|_{k-1}\right], \qquad \textbf{(S10.3)}$$

where the pre-factor, $C_0 = \frac{\epsilon_{\text{r}}\epsilon_0}{d}$, is known as the *geometric capacitance*, and the expression on the right-hand side is obtained by discretising with respect to time. Substituting these expressions into Equation **(S4.5)** yields a differential equation for the voltage drop across the diode in terms of the voltage applied to the external circuit (denoted as V_{app}), the total current density, and the combined series resistance of the circuit ($R = R_{\text{s}} + R_{\text{L}}$):

$$V_{\text{drop}}(t) + ARC_0 \frac{\partial V_{\text{drop}}(t)}{\partial t} = V_{\text{app}}(t) - J_{\text{C}}(t)AR. \quad \textbf{(S10.4)}$$

Utilising the discretised forms and making the approximation that the timestep is small enough that the conduction current changes minutely between steps ($J_{\text{C}}|_k \approx J_{\text{C}}|_{k-1}$), one finds that the voltage drop across the device at time k relates to the voltage applied to the circuit at that time, as well as the parameters of the external circuit and the current density of the previous time index, via:

$$V_{\text{drop}}\big|_k \approx \frac{V_{\text{app}}\big|_k + \frac{ARC_0}{\tau_k} V_{\text{drop}}\big|_{k-1} - J_{\text{C}}|_{k-1}AR}{1 + \frac{ARC_0}{\tau_k}}. \quad \textbf{(S10.5)}$$

Such that the boundary condition on the electric potential at the semiconductor-cathode interface (position index $N + 1$) at time t_k satisfies:

$$\psi_{N+1,k} = V_{\text{bi}} - V_{\text{drop}}\big|_k. \quad \textbf{(S10.6)}$$

We note that in the case RC effects are negligible (i.e., $R = R_{\text{s}} + R_{\text{L}} \to 0$), Equation **(S10.5)** reduces to $V_{\text{drop}}\big|_k \approx V_{\text{app}}\big|_k$, i.e., the voltage drop across the diode is, as expected, equal to the voltage applied to the external circuit.

S11. Iteration and Convergence

With the differential equations fully discretised, the iterative process described by Gummel may now be used to numerically solve them.[28] In this process, the electric potential and the electron and hole densities are assigned an iteration index l, which is iterated over until a stable solution is reached. To begin the process, some initial guesses are made for $\psi'_{j,k,l}$, $n'_{j,k,l}$, and $p'_{j,k,l}$ in the zeroth iteration ($l = 0$). The iterative process then proceeds by assuming that there exists some marginally improved values for these quantities in the iteration $l + 1$, with the potential in this iteration given by

$$\psi'_{j,k,l+1} = \psi'_{j,k,l} + (\delta\psi')_{j,k,l}, \qquad \textbf{(S11.1)}$$

where $(\delta\psi')_{j,k,l}$ is some small change that improves the potential. From Equation **(S5.19)**, an improved estimate for the electron density is then calculated using

$$n'_{j,k,l+1} = n'_{j,k,l} e^{(\delta\psi')_{j,k,l}} \approx n'_{j,k,l}\left(1 + (\delta\psi')_{j,k,l}\right), \qquad \textbf{(S11.2)}$$

where the exponential was expanded to the first order on the right hand side (assuming $\left(\delta\tilde{\psi}\right)_{j,k,l}$ is small). Similarly, an improved estimate for the hole density is

$$p'_{j,k,l+1} = p'_{j,k,l} e^{-(\delta\psi')_{j,k,l}} \approx p'_{j,k,l}\left(1 - (\delta\psi')_{j,k,l}\right). \qquad \textbf{(S11.3)}$$

Substituting Equation **(S11.2)** and **(S11.3)** into Poisson's equation gives a series of N coupled equations (encompassing all $N + 2$ points in the active layer) for the electric potential in iteration $l + 1$, in terms of the potential, and the electron and hole density at those points in iteration l (the different iterations are colour-coded for the Reader's benefit):

$$\psi'_{j+1,k,l+1} + \theta_{j,k,l}\psi'_{j,k,l+1} + \psi'_{j-1,k,l+1} = \phi_{j,k,l}, \qquad \textbf{(S11.4)}$$

where the parameters derived from the electric potential and carrier densities in iteration l are given by

$$\theta_{j,k,l} = -\left(2 + \delta'^2\left[n'_{j,k,l} + p'_{j,k,l}\right]\right), \qquad \textbf{(S11.5a)}$$

$$\phi_{j,k,l} = \delta'^2\left(n'_{j,k,l}\left[1 - \psi'_{j,k,l}\right] - p'_{j,k,l}\left[1 + \psi'_{j,k,l}\right]\right). \quad \textbf{(S11.5b)}$$

Note that the indices of the different iterations have been highlighted in different colours. From Equation **(S11.4)**, the potential at each of the points in the active layer is determined using the potential in the adjoining points. The coupled equations can therefore be expressed as a tri-diagonal matrix of the form:

$$\begin{pmatrix} \theta_{1,k,l} & 1 & 0 & \cdots & 0 & 0 & 0 \\ 1 & \theta_{2,k,l} & 1 & \ddots & 0 & 0 & 0 \\ 0 & 1 & \theta_{3,k,l} & \ddots & 0 & 0 & 0 \\ \vdots & \ddots & \ddots & \ddots & \ddots & \ddots & \vdots \\ 0 & 0 & 0 & \cdots & \theta_{N-2,k,l} & 1 & 0 \\ 0 & 0 & 0 & \cdots & 1 & \theta_{N-1,k,l} & 1 \\ 0 & 0 & 0 & \cdots & 0 & 1 & \theta_{N,k,l} \end{pmatrix} \begin{pmatrix} \psi'_{1,k,l+1} \\ \psi'_{2,k,l+1} \\ \psi'_{3,k,l+1} \\ \vdots \\ \psi'_{N-2,k,l+1} \\ \psi'_{N-1,k,l+1} \\ \psi'_{N,k,l+1} \end{pmatrix} = \begin{pmatrix} \phi_{1,k,l} - \psi'_{0,k} \\ \phi_{2,k,l} \\ \phi_{3,k,l} \\ \vdots \\ \phi_{N-2,k,l} \\ \phi_{N-1,k,l} \\ \phi_{N,k,l} - \psi'_{N+1,k} \end{pmatrix}. \quad \textbf{(S11.6)}$$

Where the boundary conditions on the potential $\psi'_{0,k}$ and $\psi'_{N+1,k}$ are known and thus independent of iteration number (though not necessarily independent of time). Such a matrix is solved using the *Thomas Algorithm*. This algorithm states that a general set of N coupled equations of the form $\alpha_i x_{i-1} + \beta_i x_i + \gamma_i x_{i+1} = \delta_i$ (with known boundary conditions x_0 and x_{N+1} that are "absorbed" into δ_1 and δ_N, respectively), are solved by rewriting the equations in the simplified form:[29]

$$x_i + \gamma''_i x_{i+1} = \delta''_i, \quad \textbf{(S11.7)}$$

where the coefficients are defined as

$$\gamma''_i = \begin{cases} \dfrac{\gamma_i}{\beta_i}, & i = 1, \\ \dfrac{\gamma_i}{\beta_i - \gamma''_{i-1}\alpha_i}, & i = 2, 3, \ldots, N, \end{cases} \quad \textbf{(S11.8)}$$

and

$$\delta''_i = \begin{cases} \dfrac{\delta_i}{\beta_i}, & i = 1, \\ \dfrac{\delta_i - \delta''_{i-1}}{\beta_i - \gamma''_{i-1}\alpha_i}, & i = 2, 3, \ldots, N. \end{cases} \quad \textbf{(S11.9)}$$

By computing these coefficients from $i = 1$ all the way up to $i = N$ (where each new set of coefficients depends on last), it follows that $x_N = \delta_N''$. Then, working backwards, the other unknowns are evaluated using $x_i = \delta_i'' - \gamma_i'' x_{i+1}$, for $i = N-1, N-2, \ldots, 1$.

After solving the tri-diagonal matrix for the discretised Poisson equation to determine $\psi'_{j,k,l+1}$, the next iteration of the electron and hole densities may now be determined. To do this, we first re-write the net generation-recombination rate seen in Equation **(S6.3)** in its normalised, discretised form (at position index j and time index k) as

$$G'_{j,k} - \mathcal{R}'_{j,k} = G'_{\text{light}}\big|_{j,k} - \gamma\left(\mu'_n|_{j,k} + \mu'_p\big|_{j,k}\right)\left(n'_{j,k}p'_{j,k} - {n'_{\text{int}}}^2\right), \quad \textbf{(S11.10)}$$

where the normalised generation rate is defined by $G'_{\text{light}}\big|_{j,k} = \frac{G_{\text{light}}|_{j,k}}{\bar{G}}$, while the normalised intrinsic carrier density is given by $n'_{\text{int}} = \frac{n_{\text{int}}}{\bar{n}}$. As the net rate given in Equation **(S11.10)** depends on both carrier densities, the iteration process may become unstable while numerically solving the continuity equations as there may be substantial variation in the densities between iterations (e.g., under illumination). To stabilize the iteration process, the continuity equations are solved in succession (as opposed to simultaneously), starting with the continuity equation for electrons:

$$a_{n,j,k,l}n'_{j-1,k,l+1} + b'_{n,j,k,l}n'_{j,k,l+1} + c_{n,j,k,l}n'_{j+1,k,l+1} = d_{n,j,k}, \quad \textbf{(S11.11)}$$

where the new coefficients are given by:

$$b'_{n,j,k,l} = b_{n,j,k,l} - \frac{1}{\tau'_k} - \gamma\left(\mu'_n|_{j,k} + \mu'_p\big|_{j,k}\right)p'_{j,k,l}, \quad \textbf{(S11.12a)}$$

$$d_{n,j,k} = -\left(G'_{\text{light}}\big|_{j,k} + \gamma\left(\mu'_n|_{j,k} + \mu'_p\big|_{j,k}\right){n'_{\text{int}}}^2 + \frac{n'_{j,k-1}}{\tau'_k}\right). \quad \textbf{(S11.12b)}$$

All the coefficients, $a_{n,j,k,l}$, $b'_{n,j,k,l}$, $c_{n,j,k,l}$, and $d_{n,j,k}$ are determined using the current iteration's $p'_{j,k,l}$, and $\psi'_{j,k,l+1}$ from the newly-solved Poisson equation, as well as $n'_{j,k-1}$ from the previous time in a transient simulation. Again, in a static simulation the timestep is effectively infinite, so the terms inversely

proportional to τ'_k vanish. As with the discretised Poisson equation, Equation **(S11.11)** can be used to write an $N \times N$-dimensional tri-diagonal matrix that can be solved (with known boundary conditions $n'_{0,k}$ and $n'_{N+1,k}$) using the Thomas algorithm, giving the next iteration of the electron current density, $n'_{j,k,l+1}$. This, in turn, can be substituted into the discretised continuity equation for holes to give

$$a_{p,j,k,l}p'_{j-1,k,l+1} + b'_{p,j,k,l}p'_{j,k,l+1} + c_{p,j,k,l}p'_{j+1,k,l+1} = d_{p,j,k}, \qquad \textbf{(S11.13)}$$

where the new coefficients are given by

$$b'_{p,j,k,l} = b_{p,j,k,l} + \frac{1}{\tau'_k} + \gamma\left(\mu'_n|_{j,k} + \mu'_p\big|_{j,k}\right)n'_{j,k,l+1}, \qquad \textbf{(S11.14a)}$$

$$d_{p,j,k} = G'_{\text{light}}\big|_{j,k} + \gamma\left(\mu'_n|_{j,k} + \mu'_p\big|_{j,k}\right){n'_{\text{int}}}^2 + \frac{p'_{j,k-1}}{\tau'_k}. \qquad \textbf{(S11.14b)}$$

Equation **(S11.13)** can now be solved using the Thomas algorithm and the boundary conditions on the hole density, giving the next iteration of the hole density ($p'_{j,k,l+1}$). This hole density is then compared with its value in the previous iteration, alongside the electron density and electric potential, to determine whether or not a convergence has been reached, i.e., whether or not the following conditions have been satisfied at each position index j:

$$\left|\frac{\psi'_{j,k,l+1} - \psi'_{j,k,l}}{\psi'_{j,k,l}}\right| < \xi, \qquad \textbf{(S11.15a)}$$

$$\left|\frac{n'_{j,k,l+1} - n'_{j,k,l}}{n'_{j,k,l}}\right| < \xi, \qquad \textbf{(S11.15b)}$$

$$\left|\frac{p'_{j,k,l+1} - p'_{j,k,l}}{p'_{j,k,l}}\right| < \xi. \qquad \textbf{(S11.15c)}$$

Here, ξ is the convergence criterion – a minimum change between iterations that must be met for convergence to be reached. We take $\xi = 10^{-6}$, indicating six-significant figure solutions, though a higher or lower accuracy can be taken.

The block diagram in **Figure S6** illustrates how the drift-diffusion model presented in this document is implemented by the *Jupyter*-based drift-diffusion tool (freely available online).[10] Once convergence has been reached, the electric potential and the carrier densities can be renormalised, and derivative parameters like the electric field and the current densities can be calculated. Furthermore, in the case of a transient simulation, the conduction current can be evaluated and used to determine the voltage drop across the diode at the next time step.

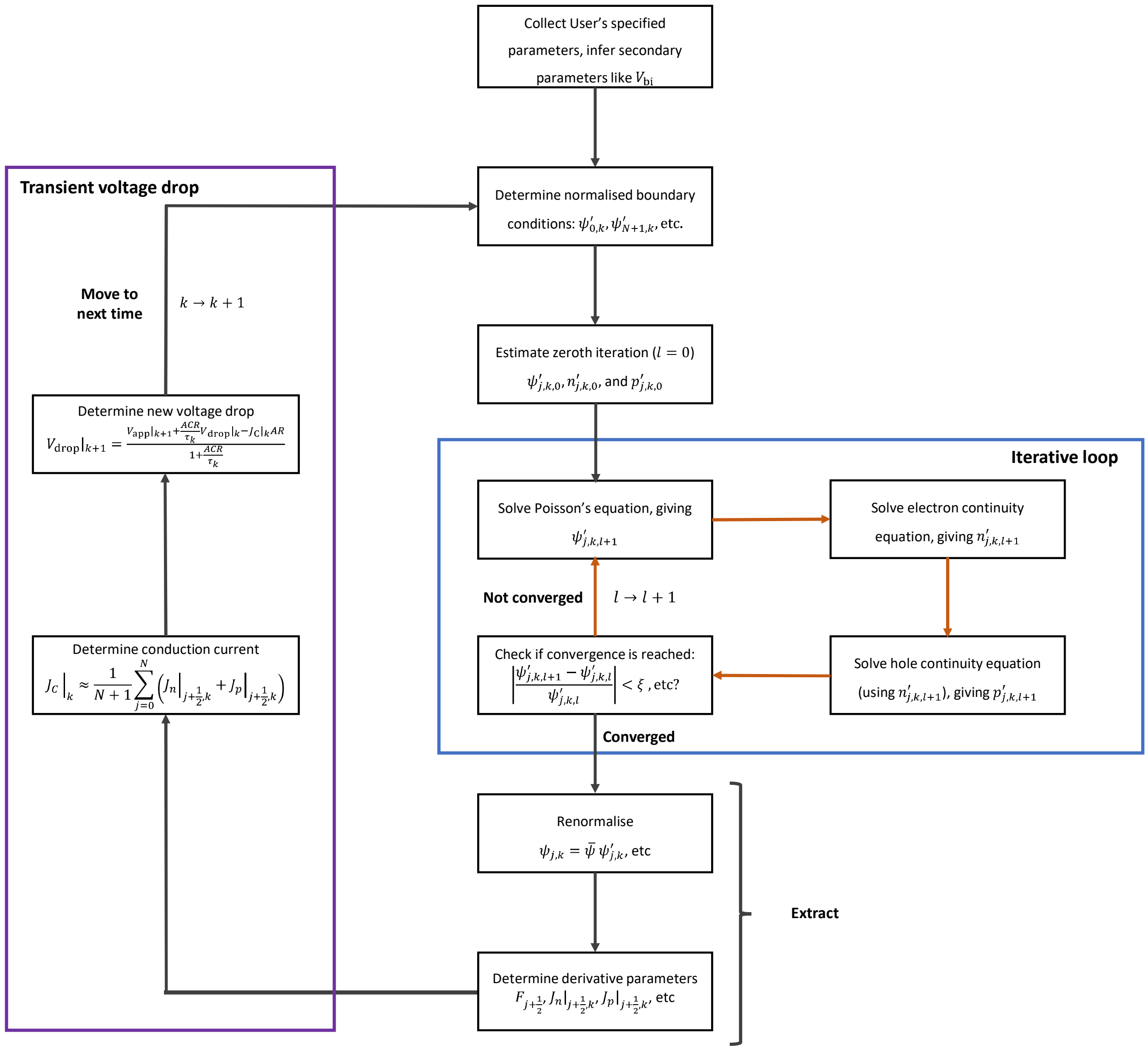

Figure S6: A block diagram illustrating the process of the numerical drift-diffusion model as implemented by the *Jupyter*-based tool freely available online,[10] from the collection of the User's parameter choices in the first step, to the iterative process used to numerically solve the equations of the drift-diffusion model, to the evaluation of the voltage drop across the diode in a transient simulation, to the ultimate extraction of the re-normalised parameters.

References

(1) Sandberg, O. J.; Tvingstedt, K.; Meredith, P.; Armin, A. Theoretical Perspective on Transient Photovoltage and Charge Extraction Techniques. *The Journal of Physical Chemistry C* **2019**, *123* (23), 14261-14271. DOI: 10.1021/acs.jpcc.9b03133.
(2) Kay, A. M. *Recombination Rate Constant Corrector*. 2023. https://github.com/Austin-M-Kay/Recombination_Rate_Constant_Corrector (accessed September 26th 2023).
(3) Hosseini, S. M.; Roland, S.; Kurpiers, J.; Chen, Z.; Zhang, K.; Huang, F.; Armin, A.; Neher, D.; Shoaee, S. Impact of Bimolecular Recombination on the Fill Factor of Fullerene and Nonfullerene-Based Solar Cells: A Comparative Study of Charge Generation and Extraction. *The Journal of Physical Chemistry C* **2019**, *123* (11), 6823-6830.
(4) Selberherr, S. *Analysis and Simulation of Semiconductor Devices*; Springer Vienna, 1984.
(5) Sandberg, O. J. Charge Collection in Thin-Film Devices Based on Low-Mobility Semiconductors: Theory, Simulation, and Applications to Organic Solar Cells. **2018**.
(6) Sze, S. M.; Li, Y.; Ng, K. K. *Physics of Semiconductor Devices*; John Wiley & Sons, Inc, 2021.
(7) Sandberg, O. J.; Nyman, M.; Österbacka, R. Effect of Contacts in Organic Bulk Heterojunction Solar Cells. *Physical Review Applied* **2014**, *1* (2), 024003. DOI: 10.1103/PhysRevApplied.1.024003.
(8) Sandén, S.; Sandberg, O. J.; Xu, Q.; Smått, J. H.; Juška, G.; Lindén, M.; Österbacka, R. Influence of Equilibrium Charge Reservoir Formation on Photo-Generated Charge Transport in TiO2/Organic Devices. *Organic Electronics* **2014**, *15* (12), 3506-3513. DOI: https://doi.org/10.1016/j.orgel.2014.09.043.
(9) Neukom, M. T.; Reinke, N. A.; Ruhstaller, B. Charge Extraction with Linearly Increasing Voltage: A Numerical Model for Parameter Extraction. *Solar Energy* **2011**, *85* (6), 1250-1256. DOI: https://doi.org/10.1016/j.solener.2011.02.028.
(10) Kay, A. M. *Drift-Diffusion Simulator*. 2023. https://github.com/Austin-M-Kay/Drift_Diffusion_Simulator (accessed September 26th 2023).
(11) Köhler, A.; Bässler, H. *Electronic Processes in Organic Semiconductors: An Introduction*; John Wiley & Sons, 2015.
(12) Griffiths, D. J. Introduction to Electrodynamics. *American Journal of Physics* **2005**, *73* (6), 574-574. DOI: 10.1119/1.4766311.
(13) Griffiths, D. J. *Introduction to Electrodynamics*; Pearson Education, Inc., 2013.
(14) Ohm, G. S. *Die Galvanische Kette: Mathematisch*; TH Riemann, 1827.
(15) Kirchhoff, G. On a Deduction of Ohm's Laws, in Connexion with the Theory of Electro-statics. *The London, Edinburgh, and Dublin Philosophical Magazine and Journal of Science* **1850**, *37* (252), 463-468.
(16) Bisquert, J. Chemical Diffusion Coefficient of Electrons in Nanostructured Semiconductor Electrodes and Dye-Sensitized Solar Cells. *The Journal of Physical Chemistry B* **2004**, *108* (7), 2323-2332. DOI: 10.1021/jp035397i.
(17) Fick, A. V. On Liquid Diffusion. *The London, Edinburgh, and Dublin Philosophical Magazine and Journal of Science* **1855**, *10* (63), 30-39.
(18) Lakhwani, G.; Rao, A.; Friend, R. H. Bimolecular Recombination in Organic Photovoltaics. *Annual Review of Physical Chemistry* **2014**, *65*, 557-581.
(19) Burke, T. M.; Sweetnam, S.; Vandewal, K.; McGehee, M. D. Beyond Langevin Recombination: How Equilibrium Between Free Carriers and Charge Transfer States Determines the Open-Circuit Voltage of Organic Solar Cells. *Advanced Energy Materials* **2015**, *5* (11), 1500123. DOI: https://doi.org/10.1002/aenm.201500123.
(20) Liu, Y.; Zojer, K.; Lassen, B.; Kjelstrup-Hansen, J.; Rubahn, H.-G.; Madsen, M. Role of the Charge-Transfer State in Reduced Langevin Recombination in Organic Solar Cells: A Theoretical Study. *The Journal of Physical Chemistry C* **2015**, *119* (47), 26588-26597. DOI: 10.1021/acs.jpcc.5b08936.

(21) Nyman, M.; Sandberg, O. J.; Österbacka, R. 2D and Trap-Assisted 2D Langevin Recombination in Polymer: Fullerene Blends. *Advanced Energy Materials* **2015**, *5* (5), 1400890.
(22) Peumans, P.; Yakimov, A.; Forrest, S. R. Small Molecular Weight Organic Thin-Film Photodetectors and Solar Cells. *Journal of Applied Physics* **2003**, *93* (7), 3693-3723. DOI: 10.1063/1.1534621 (acccessed 6/14/2023).
(23) Burkhard, G. F.; Hoke, E. T.; McGehee, M. D. Accounting for Interference, Scattering, and Electrode Absorption to Make Accurate Internal Quantum Efficiency Measurements in Organic and Other Thin Solar Cells. *Advanced Materials* **2010**, *22* (30), 3293-3297. DOI: https://doi.org/10.1002/adma.201000883.
(24) Pettersson, L. A.; Roman, L. S.; Inganäs, O. Modeling Photocurrent Action Spectra of Photovoltaic Devices Based on Organic Thin Films. *Journal of Applied Physics* **1999**, *86* (1), 487-496.
(25) Riley, K. F.; Hobson, M. P. *Essential Mathematical Methods for the Physical Sciences*; Cambridge University Press, 2011.
(26) Scharfetter, D. L.; Gummel, H. K. Large-Signal Analysis of a Silicon Read Diode Oscillator. *IEEE Transactions on Electron Devices* **1969**, *16* (1), 64-77.
(27) Jackson, J. D. Classical Electrodynamics. American Association of Physics Teachers: 1999.
(28) Gummel, H. K. A Self-Consistent Iterative Scheme for One-Dimensional Steady State Transistor Calculations. *IEEE Transactions on Electron Devices* **1964**, *11* (10), 455-465.
(29) Teukolsky, S. A.; Flannery, B. P.; Press, W.; Vetterling, W. *Numerical Recipes in C*; 1992.
