# Supplementary material for: A New Framework for Understanding Recombination-Limited Charge Extraction in Disordered Semiconductors
Source: J Phys Chem Lett. 2024 Apr 16;15(16):4416–21. doi: 10.1021/acs.jpclett.4c00218 (PMC11057038; doi:10.1021/acs.jpclett.4c00218)
Supplement: Supplementary file 2 — jz4c00218_si_002.pdf [file jz4c00218_si_002.pdf]

jz-2024-00218u.R1

Name: Peer Review Information for "A New Framework for Understanding Recombination-Limited Charge Extraction in Disordered Semiconductors"

First Round of Reviewer Comments

Reviewer: 1

Comments to the Author

This is a very conclusive study regarding the effect of NRG and of the series resistance on the accuracy of the determination of the carrier density and recombination properties of low mobility organic solar cells. In particular, I like the analytical expressions in Equations 8, 9 and 10, as they allow to correct the measured values for carrier losses due to inefficient extraction.

Therefore, I recommend publication of this work with minor revisions.

I might have missed it but what did the authors assume an electron-and hole-injection barrier in their simulations. Also, did the authors allow for surface recombination at the anode and cathode?

According to table S2, the active layer cross section is 0.1 cm2, which is a rather large area for CE or BACE experiments. In our lab, we usually use a device area of 0.01 cm2. This is to reduce the RC time, which for a typical active layer thickness of 100 nm and a series resistance (including the load resistor) of 30 Ohm would be 100 ns, but also to lower the voltage drop across the series resistance. Note that an area of 0.01 cm2 was also used in the BACE experiments by Sayed et al. Because of the large effect the device area has on the RC time and voltage drop across the series resistance, simulations for a more realistic device area should be included into the main manuscript

Can the authors please describe briefly how they took into account the reverse extraction voltage in BACE (compared to CE). Was this simply done by using a different electric field in the transit time (Eq. 5)?

The Supporting Information is divided in two parts, a description of the drift diffusion model and a second part about the theoretical modelling of charge extraction, including the analytical model to derive Equations 8-10 in the main document. While the second part indeed relates to the main document, the first part is not needed to understand the paper. If I understand this part of the SI correctly, it is a very detailed description of the theory behind the used 1D DD code and of the basic design of this code. Therefore, this first part should be moved to a second supporting document, specifically pointing out that this document refers to the 1D DD code that was used by the authors to simulate the CE experiments and that it is not needed to understand the paper.

Author's Response to Peer Review Comments:

Response Letter

A New Framework for Understanding Recombination-Limited Charge Extraction in Disordered Semiconductors

*Austin M. Kay[*1], Drew B. Riley[*1], Paul Meredith[1], Ardalan Armin[1], Oskar J. Sandberg[*2]*

[1]Sustainable Advanced Materials (Sêr-SAM), Centre for Integrative Semiconductor Materials (CISM), Department of Physics, Swansea University Bay Campus, Swansea SA1 8EN, United Kingdom

[2]Physics, Faculty of Science and Engineering, Åbo Akademi University, 20500 Turku, Finland

Email: a.m.kay.954708@swansea.ac.uk; d.b.riley@swansea.ac.uk; oskar.sandberg@abo.fi

---

We thank the Reviewer for carefully reading our work and providing helpful comments on behalf of *The Journal of Physical Chemistry Letters*. Based on these comments, we have subsequently revised and improved the manuscript. In this document, we have listed a detailed point-by-point response to address the Reviewer's comments in blue. The revisions to the manuscript are shown in red with the corresponding changes indicated in **bold**.

Reviewer's Summary:

This is a very conclusive study regarding the effect of non-geminate recombination (NGR) and of the series resistance on the accuracy of the determination of the carrier density and recombination properties of low mobility organic solar cells. In particular, I like the analytical expressions in Equations **(8)**, **(9)**, and **(10)**, as they allow to correct the measured values for carrier losses due to inefficient extraction. Therefore, I recommend publication of this work with minor revisions.

We thank the Reviewer for carefully considering our work and providing extremely useful suggestions; we have addressed all of these in the following response.

Reviewer's First Comment:

I might have missed it but what did the authors assume an electron-and hole-injection barrier in their simulations. Also, did the authors allow for surface recombination at the anode and cathode?

We assumed that the contacts were Ohmic with negligible injection barriers. Furthermore, infinite surface recombination velocities were assumed at the contacts, meaning that surface recombination is accounted for in the simulations. However, for the parameters considered in this work (Ohmic contacts and relatively large bimolecular recombination constants), the effect of surface recombination is negligible. The parameters used in the simulation are shown in Table S1 (previously Table S2) in the Supporting Information. To make this abundantly clear, the following changes were made in the paragraph preceding Figure 2 in the main text:

The results for the case of negligible series resistance ($R = R_s + R_L = 0$)**, simulated** using the parameters summarized in Table S**1**~~2~~ in the Supporting Information**,** are illustrated in Figure 2a. Therein, the input recombination coefficient was fixed at $\beta = 1.20 \times 10^{-11} \mathrm{cm}^3\mathrm{s}^{-1}$, corresponding to the case of a purely bimolecular recombination rate ($\delta = 2$). **For all results presented in this work, Ohmic contacts were assumed, such that the effects of surface recombination are negligible.** From Figure 2a…

Reviewer's Second Comment:

According to **Table S2**, the active layer cross section is $0.1\ \mathrm{cm}^2$, which is a rather large area for CE or BACE experiments. In our lab, we usually use a device area of $0.01\ \mathrm{cm}^2$. This is to reduce the RC time, which for a typical active layer thickness of 100 nm and a series resistance (including the load resistor) of 30 Ohm would be 100 ns, but also to lower the voltage drop across the series resistance. Note that an area of $0.01\ \mathrm{cm}^2$ was also used in the BACE experiments by Hosseini et al. Because of the large effect the device area has on the RC time and voltage drop across the series resistance, simulations for a more realistic device area should be included into the main manuscript.

We thank the Reviewer for highlighting this point. To demonstrate how the RC time affects n_{CE} at light intensities in the vicinity of one-Sun, and to ensure the model was applicable to larger device areas, we

selected $A = 0.1\ \mathrm{cm}^2$. In response to the Reviewer's comment, we have added a new figure (**Figure S1)** to the **Supporting Information**, in which the investigation presented for device area $A = 0.1\ \mathrm{cm}^2$ in **Figure 3** of the main text is repeated for devices with area $A = 0.01\ \mathrm{cm}^2$ and $A = 1.00\ \mathrm{cm}^2$. The following reference to the new figure was added to the main text:

As demonstrated by the simulations shown in Figure 3a, **which were simulated using device area $A = 0.1\ \mathrm{cm}^2$,** increasing the series resistance leads to reduced CE at higher light intensities. This, in turn, leads to an apparent increase in the bimolecular recombination rate constant, as shown in Figure 3b. This culminates in an overestimation of the reaction order, as illustrated by the curves in Figure 3c and 3d. **The influence of RC effects is explored further for varied *A* in Figure S1 of the Supporting Information.** Primarily, this effect…

Where the following figure was added to the Supporting Information:

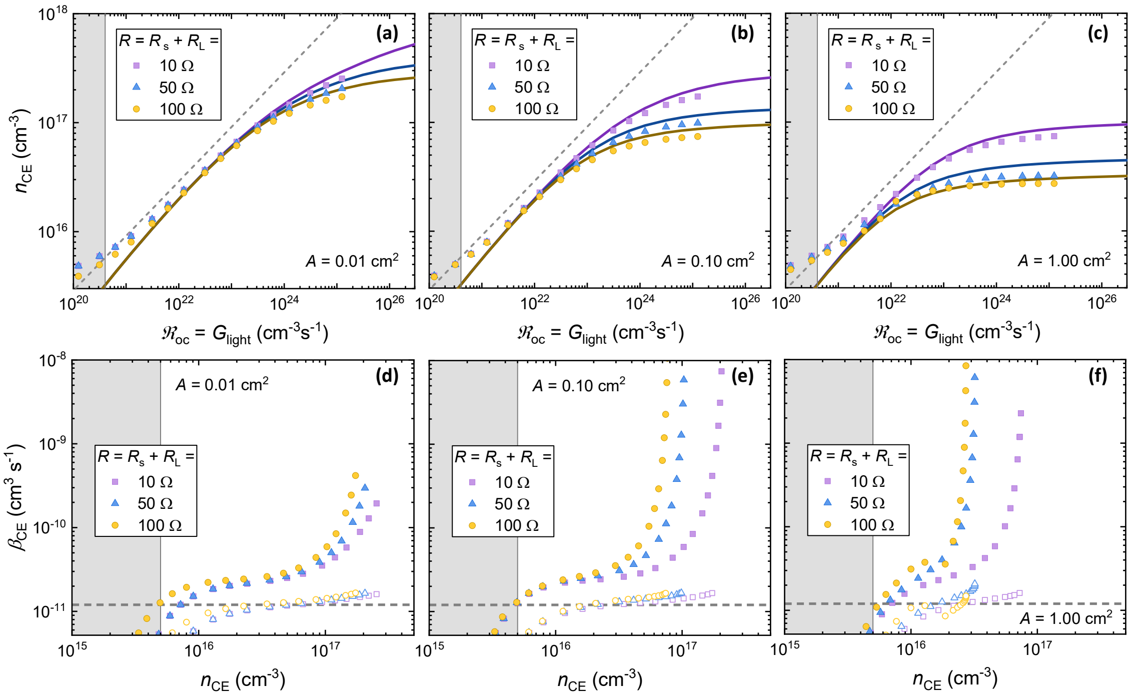

In addition, Figure 3's caption was updated to read as:

Simulated CE measurements **for a device with area $A = 0.1\ \mathrm{cm}^2$,** where…

Reviewer's Third Comment:

Can the authors please describe briefly how they took into account the reverse extraction voltage in BACE (compared to CE). Was this simply done by using a different electric field in the transit time (Equation **(5)**)?

We thank the Reviewer for highlighting this point and we recognise that we may have not emphasized it strongly enough in the manuscript. The Reviewer is correct that a different electric field is used for the transit time in case of BACE. In general, neglecting effects of series resistance, we assume a collection field given by $F = \frac{|\Delta V_{\mathrm{app}}|}{d}$, where ΔV_{app} is the difference between the open-circuit voltage and the applied (reverse) collection bias. For CE, this reduces to $F = \frac{V_{\mathrm{oc}}}{d}$. To make it clearer that the model was knowingly applied to BACE measurements, the following change was made to the discussion surrounding Figure 4:

While one blend used ITIC as the acceptor, the other used PCBM (for chemical definitions, see Table S2 in the Supporting Information).[10] **Note that a non-zero voltage bias was accounted for in the following calculations (see Section S2 of the Supporting Information).** By developing and applying an open-source computational tool that…

Reviewer's Fourth Comment:

The Supporting Information (S.I.) is divided in two parts, a description of the drift diffusion model and a second part about the theoretical modelling of charge extraction, including the analytical model to derive Equations **(8-10)** in the main document. While the second part indeed relates to the main document, the first part is not needed to understand the paper. If I understand this part of the S.I. correctly, it is a very detailed description of the theory behind the used 1D DD code and of the basic design of this code. Therefore, this first part should be moved to a second supporting document, specifically pointing out that this document refers to the 1D DD code that was used by the authors to simulate the CE experiments and that it is not needed to understand the paper.

The Reviewer is absolutely correct, Part I of the Supporting Information was originally intended to be a detailed description of the drift-diffusion model used to support this work. Although, the drift-diffusion model is not needed to understand the content of the paper, we feel it is a useful asset to present alongside our findings. We have therefore swapped the ordering of the drift diffusion model and the additional, CE-specific theoretical modelling sections, with the latter now being Part I of the Supporting Information. All section headings, equation, table, and figure numbers, and references etc throughout the Supporting Information and related cross references in the main text have been updated accordingly.

Reviewer's Fifth Comment:

According to Eq. **(8)**, the "measured BMR coefficient (based in the extracted carrier density) needs to be corrected by a factor $\left(\frac{b}{\ln(1+b)}\right)^2$ to arrive at the correct recombination coefficient. For $b \ll 1$, this factor approaches unity. However, **Figure 2b** and **Figure 4** suggest that the measured NGR coefficient is off by an almost constant factor of ca. 1.7 for small carrier densities ($b < 1$). Please explain.

We thank the Reviewer for highlighting this point and have added some discussion on this matter to the manuscript. We believe this deviation mainly arises from the fact that the carrier profiles, even at relatively high intensities, are not entirely uniform. This is in contrast with the key assumption of a homogeneous carrier density in the analytical model. At low intensities the carrier profiles are highly non-uniform, resulting in a capacitive regime (independent of bimolecular recombination). At high enough intensities, the carrier density profiles inside the active layer eventually become uniform; however, depending on the intensity, the profiles are still non-uniform near the contacts (where majority carriers dominate). After closer inspection, it turns out that this effect can be accounted for (at high enough intensities) by replacing n_{oc} with n'_{oc}, where:

$$n'_{\mathrm{oc}} \approx \frac{n_{\mathrm{oc}}}{1 + \frac{2L_D}{d}}$$

with $L_D = \sqrt{2\epsilon_r \epsilon_0 k_B T/(q^2 n_{oc})}$ being the associated Debye screening length. Note that $n'_{oc} \to n_{oc}$ at very high intensities. To account for this, the following paragraph was added between Equation **(6)** and Equation **(7)** in the main text:

Since uniform carrier distributions are assumed at $t = 0$, in the absence of recombination the extracted charge carrier density becomes $n_{CE} = n_{oc}$ in this case. For ohmic contacts, however, there is generally a considerable energy level bending near the contact, suppressing the electron (hole) density near the anode (cathode) in this region. The width of this region is approximately given by the associated Debye screening length $L_D = \sqrt{2\epsilon_r \epsilon_0 k_B T/(q^2 n_{oc})}$.[29] Accounting for this effect, we find that n_{oc} can be approximated by $n'_{oc} \approx n_{oc}/\left(1 + \frac{2L_D}{d}\right)$ for small L_D.

Where reference 29 is *Relating Charge Transport, Contact Properties, and Recombination to Open-Circuit Voltage in Sandwich-Type Thin-Film Solar Cells* by Sandberg et al. (https://journals.aps.org/prapplied/abstract/10.1103/PhysRevApplied.5.044005). In addition, the following adjustment was made to the discussion surrounding Equation **(8)**:

Hence, the extracted carrier density may be written as (see **Section S1** of the **Supporting Information**):

$$n_{CE} = \cancel{n_{oc}} \cancel{\times \frac{\ln(1+b)}{b}} \frac{1}{\beta t_{tr}} \ln\left(1 + \frac{\beta n_{oc} t_{tr}}{1 + \frac{2L_D}{d}}\right). \quad (8)$$

where ~~$b \equiv$~~ $\beta n_{oc} t_{tr}$ **is the ratio between the transit time and the carrier lifetime**, which depends on the intensity via n_{oc} through Equation **(5)**. **Note that, within the logarithm, we have corrected n_{oc} for the Debye screening length to (partially) account for the effect of Ohmic contacts.** Accordingly…

Where the changes made in **green** indicate additions made in response to this comment. The addition of this factor could explain the observed deviation between the input bimolecular recombination rate and the corrected bimolecular recombination that the Reviewer has noted in **Figure 2b**. The following changes were made to the discussion surrounding **Figure 2b** to acknowledge this fact:

While the β'_{CE} values determined using Equation **(9)** are far more accurate than the β_{CE} values determined using Equation **(4)**, there are still fluctuations in the corrected data points; these are likely due to additionally

complexities, such as **inhomogeneities in the carrier density profiles** ~~**the diffusion of charge carriers,**~~[6] ~~**that**~~ **which** are neglected in the analytical model…

Where reference number 6 points to *Meaning of Reaction Orders in Polymer:Fullerene Solar Cells* (Phys. Rev. B **86**, 165201, **2012**) by Thomas Kirchartz and Jenny Nelson, as the Reviewer recommends in their next comment. All figures have been updated accordingly, with the effect of the Debye correction factor on Figure 2a illustrated below (for the benefit of the Reviewer):

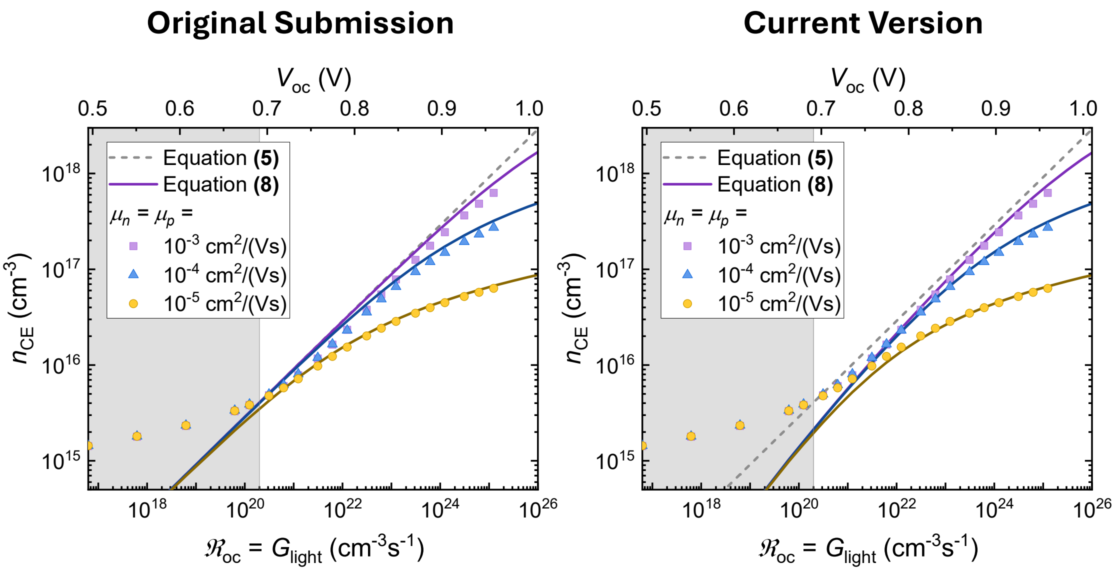

Finally, Equation **(9)** was updated in accordance with the above changes:

$$\beta'_{\mathrm{CE}} = \beta_{\mathrm{CE}} \times \left[\cancel{\frac{\ln(1+b)}{b}} \frac{1}{\beta n_{\mathrm{oc}} t_{\mathrm{tr}}} \ln\left(1 + \frac{\beta n_{\mathrm{oc}} t_{\mathrm{tr}}}{1 + \frac{2L_{\mathrm{D}}}{d}}\right)\right]^2 \qquad \textbf{(9)}$$

Reviewer's Sixth Comment:

The author fit the experimental β_{CE} (n_{CE}) for the NT812:ITIC and NT812:PCBM blend, thereby choosing t_{tr} and β to arrive at a carrier density independent recombination coefficient. However, for an active layer thickness of 100 nm and a realistic BACE collection bias of -3V, the given transient time of 2.37×10^{-6} s translates into $\mu_n + \mu_p = 10^{-5}$ cm^2/Vs. This is almost two orders of magnitude smaller than the sum of the mobilities from SCLC. Furthermore, it should be noted that according to the RPV measurements in Figure

S3 of the aforementioned paper (performed at SC), 80% of the photogenerated carriers left the device within 300 µs. Therefore, an extraction time as large as 2.4 µs, as deduced from the fit of the density dependent recombination coefficient in the present submission, is highly unlikely. The same applies to the NT812:PCBM blend, which according to the text in the main document of Mehrdad et al. has an even higher $\mu_n + \mu_p$ of 2.4×10^{-3} cm²/Vs. For a 100 nm layer thickness and a -3V collection bias, an effective extraction time of 10 ns is predicted, which is a factor of 340 faster than the value given in the text (if I am not mistaken). Therefore, other reasons than recombination during extraction must account for the higher order recombination in these systems. For the NT812:ITIC blend, a possible reason may be a broad possibly exponential trap distribution. Recent work by Shoaee et al reported a very broad DOS for this blend [10.1002/aenm.202203576]. Another possible reason can be an inhomogeneous steady state carrier profile as outlined by Kirchartz [10.1103/PhysRevB.86.165201].

We thank the Reviewer for bringing this to our attention. We believe that both a misconception on the Reviewer's part and two errors on our part must be unpacked from this comment. To begin, the time that the Reviewer quotes from the manuscript (2.37×10^{-6} s for NT812:ITIC, and 3.55×10^{-6} s for NT812:PCBM) is not the transit time (t_{tr}), but an *effective* transit time $t_{\mathrm{tr}}^{\mathrm{eff}}$ which also accounts for the external series resistance, i.e., $t_{\mathrm{tr}}^{\mathrm{eff}} = \sqrt{2RCAt_{tr}\beta_L/\beta} = \sqrt{\frac{2qRA}{F\beta}}$. Note that this quantity is independent of the mobilities. In the model presented in the original submission, the parameters β and $t_{\mathrm{tr}}^{\mathrm{eff}}$ were determined in the fitting. For the NT812:ITIC device described by Hosseini et al., $A = 0.011\ \mathrm{cm}^2$, $d = 100$ nm, and the parameters determined from the fitting were $\beta = 2.50 \times 10^{-11}\ \mathrm{cm}^3\mathrm{s}^{-1}$ and $t_{\mathrm{tr}}^{\mathrm{eff}} = 2.37 \times 10^{-6}$ s. Substituting in these values, would yield $R \sim 10\ \mathrm{k\Omega}$, which is two orders of magnitude larger than expected.

We agree with the Reviewer that other factors may be limiting the analytical model. In particular, inhomogeneous carrier densities prior to charge extraction that can be expected a lower light intensities, while the analytical model assumes homogenous carrier densities at time $t = 0$. We believe, however, that the problem likely arises in approximations made during the derivation of the analytical model. Which brings us to the first error on our part. In the original derivation of the analytical model, we assumed the

recombination lifetime to be much smaller than the RC time constant, i.e., $\tau_\beta = \frac{1}{\beta n_{oc}} < \tau_{RC} = ARC_0$ (where $C_0 = \frac{\epsilon_r \epsilon_0}{d}$ is the geometric capacitance, A is the area, and R is the resistance). However, this assumption is generally only valid for high intensities. We have accordingly revised the derivation and have adjusted the discussion in **Section S1** of the **Supporting Information** to read as:

After combining these together, ~~for $\frac{1}{\beta n_{oc}} \ll RC$~~ the following **expression** ~~**approximation**~~ can ~~**then**~~ be obtained **for the sum of the normalised depletion widths**:

$$\frac{l_n(t) + l_p(t)}{d} \approx \frac{\beta t^2}{2 t_{tr} ARC_0 \beta_L} \equiv \frac{t^2}{{t_{tr}^{eff}}^2} = \frac{t}{t_{tr}} - \frac{\beta_L}{\beta}\frac{\tau_{RC}}{t_{tr}} \ln\left[1 + \frac{t}{\tau_\beta + \frac{\beta_L}{\beta}\tau_{RC}}\right] \qquad \text{(S1.12)}$$

where $\tau_{RC} = ARC_0$ **is the lifetime associated with RC effects and** $\tau_\beta = \frac{1}{\beta n'_{oc}}$ **is the lifetime associated with the recombination process. We note that Equation (S1.12) reduces to the expected expression in the limit that $R \to 0$ (giving $\tau_{RC} \to 0$).** The effective transit time **(t_{tr}^{eff}) at which recombination ceases** in **this** ~~**the**~~ RC-dominated limit (**defined by** ~~**at which**~~ $l_n(t_{tr}^{eff}) + l_p(t_{tr}^{eff}) \equiv d$) **can be determined numerically as the root of the expression**

$$t_{tr}^{eff} - \frac{\beta_L}{\beta}\tau_{RC} \ln\left[1 + \frac{t_{tr}^{eff}}{\tau_\beta + \frac{\beta_L}{\beta}\tau_{RC}}\right] - t_{tr} = 0, \qquad \text{(S1.13)}$$

~~**is given by $t_{tr}^{eff} = \sqrt{2ARC_0\beta_L t_{tr}/\beta}$, while**~~ **noting that** t_{tr} is defined by Equation **(S1.7)** as the corresponding time in the low-resistance case. **Using a second-order Taylor expansion of the natural logarithm, an approximation for t_{tr}^{eff} may be written as**

$$t_{tr}^{eff} \approx \left(1 + \frac{\tau_\beta}{\tau'_{RC}}\right)\left(-\tau_\beta + \sqrt{\tau_\beta^2 + 2\tau'_{RC} t_{tr}}\right), \qquad \text{(S1.14)}$$

where $\tau'_{RC} = \frac{\beta_L}{\beta}\tau_{RC}$ has been defined to simplify notation. We find that this approximation is only valid for low-to-moderate light intensities (less than around 10 Suns). Using the effective transit time ~~**Accordingly**~~, the extracted charge carrier density **can be calculated using** ~~**takes the form**~~

$$\frac{n_{\mathrm{CE}}}{n'_{\mathrm{oc}}} = \mathbf{1}\cancel{n_{\mathrm{oc}}} - \frac{1}{n'_{\mathrm{oc}}}\int_0^{t_{\mathrm{tr}}^{\mathrm{eff}}} \bar{\mathcal{R}}_{\mathrm{CE}}(t)\,\mathrm{d}t$$

$$= 1 - \frac{\tau_\beta}{t_{\mathrm{tr}}}\int_0^{t_{\mathrm{tr}}^{\mathrm{eff}}} \frac{1}{(\tau_\beta + t)^2}\left(t_{\mathrm{tr}} - t + \frac{\beta_{\mathrm{L}}}{\beta}\tau'_{\mathrm{RC}}\ln\left[1 + \frac{t}{\tau_\beta +}\right]\right) \qquad \textbf{(S1.15)}$$

$$\cancel{n_{\mathrm{oc}}\left[1 - \beta n_{\mathrm{oc}}\int_0^{t_{\mathrm{tr}}^{\mathrm{eff}}}\left(1 - \frac{t^2}{t_{\mathrm{tr}}^{\mathrm{eff}\,2}}\right)\frac{\mathrm{d}t}{[1+\beta n_{\mathrm{oc}}t]^2}\right]}.$$

After evaluating the integral, we find **that the ratio of the extracted carrier density and the open-circuit carrier density can be written as:**

$$\frac{n_{\mathrm{CE}}}{n'_{\mathrm{oc}}} = \frac{\tau_\beta}{t_{\mathrm{tr}}}\left(\frac{t_{\mathrm{tr}} - t_{\mathrm{tr}}^{\mathrm{eff}}}{t_{\mathrm{tr}}^{\mathrm{eff}} + \tau_\beta}\right) + \frac{\tau_\beta}{t_{\mathrm{tr}}}\left(1 + \frac{\tau'_{\mathrm{RC}}}{\tau_\beta + t_{\mathrm{tr}}^{\mathrm{eff}}}\right)\ln\left(1 + \frac{t_{\mathrm{tr}}^{\mathrm{eff}}}{\tau_\beta + \tau'_{\mathrm{RC}}}\right), \qquad \textbf{(S1.16)}$$

~~where $b_{\mathrm{eff}} \equiv \beta n_{\mathrm{oc}} t_{\mathrm{tr}}^{\mathrm{eff}}$~~ **which reduces to Equation (S1.8) in the limit that $R \to 0$ (as $\tau_{\mathrm{RC}} \to 0$ and $t_{\mathrm{tr}}^{\mathrm{eff}} \to t_{\mathrm{tr}}$), as expected.**

Correspondingly, we have the updated the discussion surrounding Equation **(10)** and **Figure 4** of the main text to read as:

In the limit that resistive (RC) losses dominate**, such that the RC time $\tau_{\mathrm{RC}} = ARC \neq 0$**, we find that the extracted carrier density and bimolecular recombination rate constant, denoted as $n_{\mathrm{CE}}^{\mathrm{RC}}$ and $\beta_{\mathrm{CE}}^{\mathrm{RC}}$, respectively, can be analytically approximated with (see **Section S2 of the Supporting Information**)

$$n_{\mathrm{CE}}^{\mathrm{RC}} = n_{\mathrm{oc}} \times f\cancel{\frac{2}{b_{\mathrm{eff}}}\left[1 - \frac{\ln(1+b_{\mathrm{eff}})}{b_{\mathrm{eff}}}\right]}, \qquad \textbf{(10a)}$$

$$\beta_{\mathrm{CE}}^{\mathrm{RC}} = \beta_{\mathrm{CE}} \times f^2\cancel{\frac{4}{b_{\mathrm{eff}}^2}\left[1 - \frac{\ln(1+b_{\mathrm{eff}})}{b_{\mathrm{eff}}}\right]^2}, \qquad \textbf{(10b)}$$

$$f = \frac{1}{\beta n_{\mathrm{oc}} t_{\mathrm{tr}}}\left[\frac{t_{\mathrm{tr}} - t_{\mathrm{tr}}^{\mathrm{eff}}}{\tau_\beta + t_{\mathrm{tr}}^{\mathrm{eff}}} + \left(1 + \frac{\frac{\beta_{\mathrm{L}}}{\beta}\tau_{\mathrm{RC}}}{\tau_\beta + t_{\mathrm{tr}}^{\mathrm{eff}}}\right)\ln\left(1 + \frac{t_{\mathrm{tr}}^{\mathrm{eff}}}{\tau_\beta + \frac{\beta_{\mathrm{L}}}{\beta}\tau_{\mathrm{RC}}}\right)\right], \qquad \textbf{(10c)}$$

where **f is a correction factor,** ~~$b_{\mathrm{eff}} = \frac{t_{\mathrm{tr}}^{\mathrm{eff}}}{\tau_\beta}$, with $t_{\mathrm{tr}}^{\mathrm{eff}} = \sqrt{2ARCt_{\mathrm{tr}}\beta_L/\beta}$ being an effective transit time~~, **$\tau_\beta = \left(1 + \frac{2L_{\mathrm{D}}}{d}\right)/\beta n_{\mathrm{oc}}$ is the recombination lifetime (including the Debye screening factor),** $\beta_{\mathrm{L}} =$

$q(\mu_n + \mu_p)/\epsilon_r\epsilon_0$ **is** ~~**and**~~ the Langevin recombination rate constant ~~**being defined as**~~, **and t_{tr}^{eff} is the effective transit time at which recombination stops, which can be approximated for light intensities below 10 Suns (see Supporting Information) with**

$$t_{tr}^{eff} \approx \left(1 + \frac{\tau_\beta}{\frac{\beta_L}{\beta}\tau_{RC}}\right)\left(\sqrt{\tau_\beta^2 + \frac{2\beta_L}{\beta}\tau_{RC}t_{tr}} - \tau_\beta\right). \quad (11)$$

As expected, in the limit that $R \to 0$ the analytical expressions for the extracted carrier density and the bimolecular recombination rate constant given in Equation (10) reduce to Equation (8) and (9), respectively. Equation **(10)** was…

With the updated model for CE in the case of non-negligible resistance in hand, we have revised Figure 3. For the benefit of the Reviewer, we have compared both the old and new models for the R-dominated CE process in the same graph below:

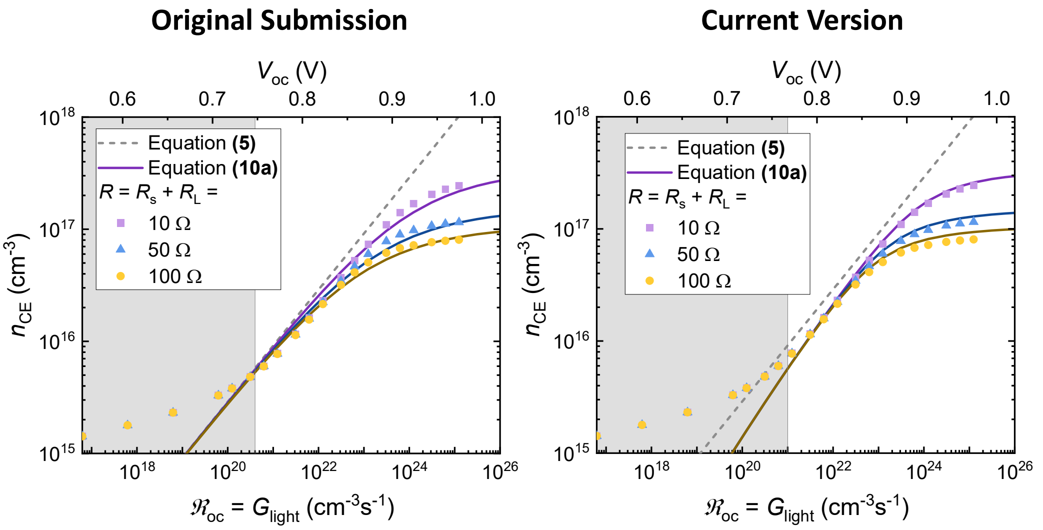

Note that the onset of the capacitive region has shifted to *higher* light intensities (around 0.1 Suns) in the new model.

This brings us to the second error on our part. To correct the experimental data measured by Hosseini et al., we created a computational tool that employs a method-of-least-squares method to determine the fitting parameters: the bimolecular recombination rate constant (β) and the effective transit

time ($t_{\mathrm{tr}}^{\mathrm{eff}}$). For this fitting, we had previously assumed that the transit time is *independent* of the light intensity/generation rate, i.e., that the electric field F is constant. However, since t_{tr} is inversely proportional to $F = \frac{V_{\mathrm{oc}}+V_{\mathrm{bias}}}{d}$, then we now account for the light-intensity dependence of the generation rate by writing the open-circuit voltage as

$$V_{\mathrm{oc}} = V_{\mathrm{oc}}^{\odot} + \frac{k_{\mathrm{B}}T}{q}\ln\left(\frac{G_{\mathrm{light}}}{G_{\mathrm{light}}^{\odot}}\right),$$

where $V_{\mathrm{oc}}^{\odot}$ and $G_{\mathrm{light}}^{\odot}$ are the open-circuit voltage under one-Sun conditions.

In accordance with all the above changes, we have adjusted the discussion surrounding Figure 4. In light of the new model for n_{CE}, it appears that the data was measured at light intensities in the vicinity of the capacitive regime. Ideally, the measurements would need to extend to around 10-Suns. To try to correct this data, we have therefore assumed a large $R = 1\ \mathrm{k\Omega}$ to inflate the RC time. In the presence of a large R, the effect voltage drop across the device for $t \ll \tau_{\mathrm{RC}}$ may be approximated with

$$V \approx \frac{V_{\mathrm{oc}} + V_{\mathrm{bias}}}{1 + \frac{\beta_{\mathrm{L}}\tau_{\mathrm{RC}}n_{\mathrm{oc}}}{1 + \frac{2L_{\mathrm{D}}}{d}}}.$$

The updated Figure 4 is:

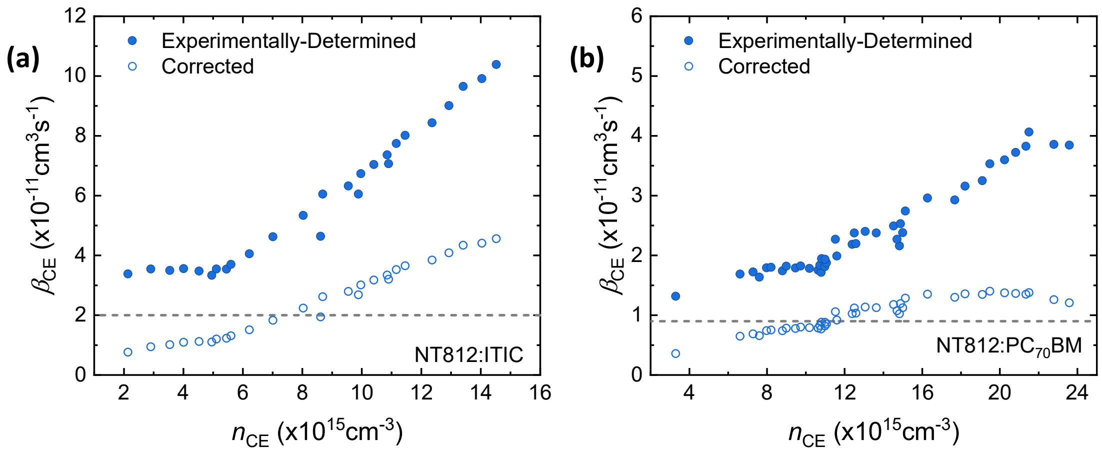

And the following revisions were made to the discussion surrounding Figure 4:

1. Finally, to demonstrate **how high resistances can lead to** ~~**the importance of accounting for**~~ incomplete CE **and possibly an apparent carrier density-dependence in the obtained bimolecular recombination rate constant**, we applied…
2. By developing and applying an open-source computational tool that employs a least-squares method to estimate β ~~**and** $t_{\mathrm{tr}}^{\mathrm{eff}}$~~(available as **Supplemental Material**),[30] we found **that** the **carrier density-dependency observed in the** $\boldsymbol{\beta_{\mathrm{CE}}}$ **data** ~~**effective transit time and corrected bimolecular recombination rate in NT812:ITIC to be** $t_{\mathrm{tr}}^{\mathrm{eff}} \approx 2.37 \times 10^{-6}$ **s and** $\beta = 2.50 \times 10^{-11}$ **cm³s⁻¹, respectively. For NT812:PC₇₀BM, on the other hand, these quantities were found to be** $t_{\mathrm{tr}}^{\mathrm{eff}} \approx 3.55 \times 10^{-6}$ **s and** $\beta = 9.89 \times 10^{-12}$ **cm³s⁻¹. As illustrated**~~ **plotted** in Figure 4 **can be corrected for if** $\boldsymbol{R = 1000\ \Omega}$, ~~**these estimates can be used to correct the apparent increase in the obtained bimolecular recombination rate constant at higher light intensities,**~~ suggesting that such variations ~~**are likely due to**~~ **could be described by** incomplete CE, rather than higher-order ($\delta > 2$) recombination processes. **The trend in** $\boldsymbol{\beta'_{\mathrm{CE}}}$ **versus** $\mathbf{n_{CE}}$ **observed in Figure 4 now reflects that seen in Figure 2b and Figure 3b**. **In particular, the large increase in measured** $\boldsymbol{\beta_{\mathrm{CE}}}$ **has been tempered with a smaller rise, followed by a fall. Note that the parameters used to correct this data are provided in Table S3 in the Supporting Information, alongside an expression for the resistance-dominated voltage in the limit that** $\boldsymbol{t \ll \tau_{\mathrm{RC}}}$**.**
3. The empty data points indicate the bimolecular recombination rate constants ~~**determined using the computational tool we provide as Supporting Information,**[30] **which determined for**~~ **corrected using** $\boldsymbol{R = 1000\ \Omega}$ ~~**for the NT812:ITIC device that** $t_{\mathrm{tr}}^{\mathrm{eff}} = 2.37 \times 10^{-6}$**s and** $\beta = 2.50 \times 10^{-11}$**cm³s⁻¹. Whereas for the NT812:PC₇₀BM device, these were found to be** $t_{\mathrm{tr}}^{\mathrm{eff}} = 3.55 \times 10^{-6}$**s and** $\beta = 9.89 \times 10^{-12}$**cm³s⁻¹.**~~ The corrected recombination rates are indicated by the dashed grey lines in both panels.

To complement these changes, the following revisions were made to the:

- Abstract: To overcome this intrinsic limitation of the CE experiment, we present an analytical model that accounts for charge carrier recombination, ~~**before validating**~~ **validate** it using numerical simulations, and **employ** ~~**using**~~ it to correct **the carrier density-dependence observed in** experimentally-determined bimolecular recombination rates
- Introduction: We then validate this model against data simulated using the drift-diffusion model, before demonstrating how the model can be used to correct **the carrier density-dependence observed** in experimental~~**ly-determined**~~ data.
- Conclusion: We have ~~**applied**~~ **demonstrated how** ~~**these to correct**~~ **this model can applied by correcting the carrier density-dependence of** published bimolecular recombination rate data for OPV devices ~~**with NT812:ITIC and NT812:PCBM active layers**~~, demonstrating that the technique and computational tool can indeed be used to re-interpret existing measurements in the literature.

Reviewer's Seventh Comment:

A minor point: the authors may wish to add that the parameter b is equal to the ratio of the transient time and the carrier lifetime.

We chose to write the correction factor in terms of b to explicitly show the dependence of n_{CE} on n_{oc} and β. T make this clearer while also acknowledging that b is equal to the ratio of the transit time and the carrier lifetime, we have made the following changes to the discussion surrounding Equation (7) and Equation (8) accordingly (where the changes illustrated in **green** are in response to this comment):

…this leads to a time-dependent carrier density of the form

$$n(t) = p(t) = \frac{n_{\mathrm{oc}}}{1 + \beta n_{\mathrm{oc}} t}, \quad \textbf{(7)}$$

where $\frac{1}{\beta n_{\mathrm{oc}}}$ corresponds to the **carrier** lifetime associated with the bimolecular recombination process. Hence, the extracted carrier density may be written as **(see Section S1 of the Supporting Information)**:

$$n_{\mathrm{CE}} = \cancel{n_{\mathrm{oc}} \times} \frac{\cancel{\ln(1+b)}}{\cancel{b}} \frac{\mathbf{1}}{\boldsymbol{\beta t_{tr}}} \ln\left(\mathbf{1} + \frac{\boldsymbol{\beta n_{\mathrm{oc}} t_{tr}}}{\mathbf{1} + \frac{\mathbf{2}\boldsymbol{L_{\mathrm{D}}}}{\boldsymbol{d}}}\right). \quad \mathbf{(8)}$$

where ~~b~~= $\beta n_{\mathrm{oc}} t_{\mathrm{tr}}$ **is the ratio of the transit time and the carrier lifetime,** which depends…

Additional Comments from *The Journal of Physical Chemistry Letters*:

Please address the following formatting notes before submitting your revised manuscript:

1. Provide a TOC image per journal guidelines (2 in x 2 in; on the same page as the abstract) with the heading “TOC Graphic” above the graphic. The graphic should be in the form of a structure, graph, drawing, photograph, or scheme—or a combination. Non-scientific cartoon-like images or caricatures are discouraged.

The following graphic has been prepared for the Table of Contents:

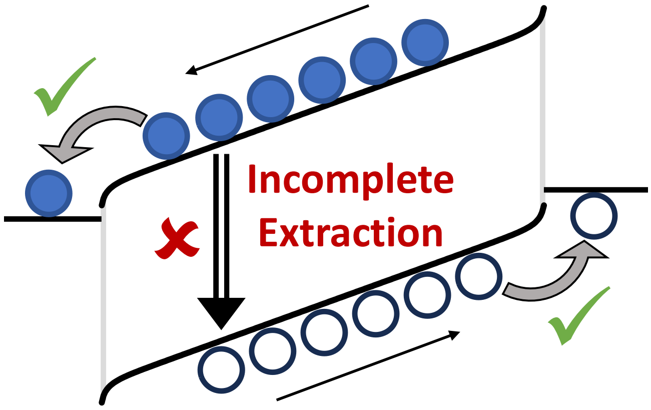

2. The supporting-information (SI) paragraph in the main file is incomplete. Please list each item (including graphics) that can be found in the SI-for-Publication file individually.

The Supporting Information paragraph have now been added as:

Supporting Information: Additional simulations and derivations, and estimation of uncertainties in the parameter extraction; a detailed description of the drift-diffusion model and an accompanying, open-source computational tool with an accessible graphical user interface.

3. In both the main file and the supporting information, fix the style of all references to use JPCL formatting (check all references carefully). ***JPC Letters reference formatting requires that journal references should contain: () around numbers; author names; article title (titles entirely in title case or entirely in lower case); abbreviated journal title (italicized); year (bolded); volume (italicized); and pages (first-last). Book references should contain author names; book title (in the same pattern); publisher; city; and year. Websites must include date of access.

We have now updated the references to *JPCL's* format accordingly.

4. Please number SI pages in the following format: "S1, S2…"

The page numbers of the Supporting Information have now been placed in this format.

5. Please submit your publication files without any markups. Please include annotated version(s) of your revised publication file(s) with colored text or highlights indicating the revisions that you have made as "Supporting Information for Review Only."

The files for review have prepared as required.
